# Employment of the fluorescent probe hydroxy-naphthyl-methylbenzoxazole-based dye and its combination with fluorescent silica nanoparticles as potential heavy metal-targeting systems: results and troubleshooting in Cd-polluted environments

**DOI:** 10.1007/s00216-026-06601-3

**Published:** 2026-06-08

**Authors:** Daniele Lopez, Mariele Montanari, Caterina Ciacci, Giovanna Panza, Eleonora Macedi, Daniele Paderni, Ludovica Di Fabrizio, Mattia Tiboni, Daniele Gori, Chiara Barattini, Angela Volpe, Alfredo Ventola, Stefano Papa, Vieri Fusi, Barbara Canonico

**Affiliations:** 1 Department of Biomolecular Sciences (DISB), University of Urbino Carlo Bo, 61029 Urbino, Italy; 2 Department of Pure and Applied Sciences (DiSPeA), University of Urbino Carlo Bo, 61029 Urbino, Italy; 3 AcZon Srl, 40050 Monte San Pietro, Italy

**Keywords:** Cadmium detection, Fluorescent dyes HNBO based, Si-NPs, Live-cell imaging, *Mytilus galloprovincialis*, Marine environment

## Abstract

To address the critical need for monitoring heavy metal (HM) contamination in living systems, this study evaluates a novel HNBO-based fluorescent probe for the detection of cadmium (Cd) in human cell lines and mussel hemocytes. Both the free probe (HNBO-DEN) and its conjugate with core-shell silica nanoparticles (HNBO-DEN-NPs) demonstrated robust Cd-sensing capabilities without compromising cellular viability. The key findings include targeted detection (the fluorescent probe successfully identified cadmium in bioindicators, providing a real-time alternative to traditional, destructive analytical techniques) and dual system efficiency: both free and silica NP-conjugated probes (HNBO-DEN-NPs) proved effective although to a different extent and with their own peculiarities: while HNBO-DEN is more effective at detecting Cd concentrations in the cytoplasm, the NP conjugate proves more suitable for detecting the metal in its target and storage organelles. The combined use of these probes may account for precise, live-cell tracking of both pollutant (Cd) and detector (NP), maximizing diagnostic, environmental, and cytological information.

**Graphical abstract:**

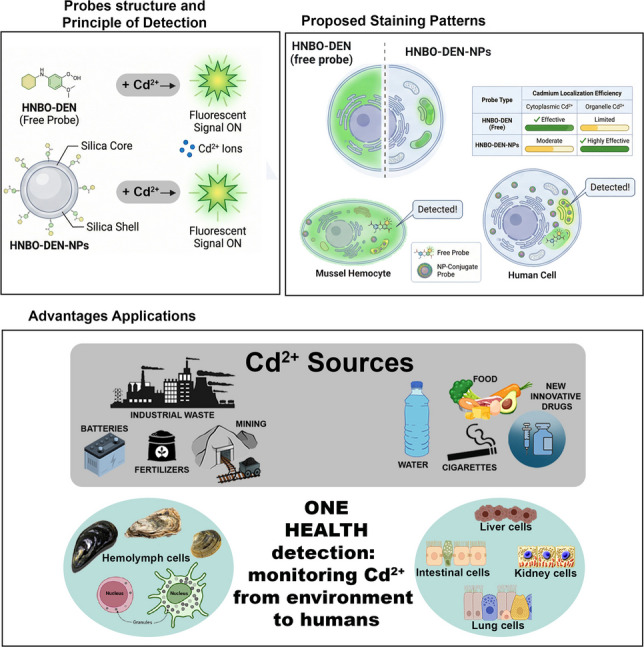

**Supplementary Information:**

The online version contains supplementary material available at 10.1007/s00216-026-06601-3.

## Introduction

Due to the growing concern about heavy metals (HMs) contamination, their selective detection has developed into an important branch of research in chemistry, biology, environmental science, agricultural science and food safety [1–3]. Among all the pollutants, HMs are extremely harmful substances, even at extremely low concentrations and cause damage to both human and environmental health. They are persistent in the ecosystem and can easily accumulate in aquatic products and other foodstuffs (in the form of ions) and be absorbed into the human body via the food chain. This gradually increases the threat to health via unintentional intake of HMs in our daily lives [4–6]. The severe toxic effects induced by HMs in the environment and in aquatic and terrestrial organisms can be manifested by significant changes in soil properties and its biological activity [7, 8]. HMs can be determined by instrumental methods such as atomic absorption spectrometry, inductively coupled plasma mass spectrometry, neutron activation analysis, anodic stripping voltammetry, X-ray fluorescence spectrometry, ion chromatography, Raman spectroscopy, electrothermal atomic absorption spectrometry, cold vapour atomic absorption spectrometry and potentiometric ion-selective electrodes [9–11]. However, all the mentioned techniques do not have the ability to be applied to an organism or a cell in its living state. In fact, these methods require expensive instruments, well-controlled experimental conditions, and time-consuming and complicated sample pretreatment procedures, lacking any other information on cell viability and cell functions. The possibility of detecting HMs in dead, apoptotic, stressed or viable cells simultaneously present in the biological samples is of great importance in managing bioindicator organisms.

Among the techniques useful for HMs detection, the employment of fluorescent small molecules (easily applicable in biological samples) has attracted more attention due to several distinct advantages in terms of (i) sensitivity, (ii) selectivity, (iii) simplicity, (iv) response time, (v) non-destructive methodology, (vi) high sampling frequency, (vii) low equipment cost, (viii) direct visual perception, (ix) easy signal detection, and (x) greater suitability for the analysis of trace metal ions in complex matrices, as highlighted by several researchers [12, 13]. Moreover, considerable efforts have been made to develop selective fluorescence chemosensors to determine toxic HMs [13–16]. The most recent research progresses on fluorescence techniques for the detection of heavy metal ions are related to the analysis of food matrices. in the specific case of cadmium, the employment of specific fluorescent sensors has the potential to elucidate the carcinogenic mechanisms of cadmium in vivo and to monitor temporal changes in cadmium concentration within the environment. Furthermore, fluorescent chemosensors for Cd^2+^ detection could serve as ideal tools for evaluating and dynamically mapping intracellular fluctuations of the metal ion employing quantitative techniques (as Flow Cytometry (FC)) or qualitative approaches (such as fluorescence and/or Confocal microscopy-FM and/or CM). The employment of these probes in HMs detection could promote advances in diagnosis, treatment and environmental monitoring, since they can provide accurate contamination detection [17]: nowadays, the practical applications, including in vivo imaging, of some fluorescent chemosensors remain limited, primarily due to their poor water solubility or pH-dependent fluorescence in the different biological contexts [18]. Furthermore, most of these fluorescent molecules are mainly revealed through spectroscopic analyses and cannot furnish information on HMs in viable cells and their compartments. These aspects encouraged the research of new and better performing sensors. Searching for probes suitable for pollutants detection in ecological and environmental fields, many research laboratories have developed their own in-house fluorescent probes for the detection of HMs [12, 19–21], which are more or less patented and waiting to be commercialized for reproducible and reliable use (Table S1) Among commercial fluorescent dyes for the detection of HMs, Leadmium Green™ (LG) is a small molecule fluorescent sensor for the detection of free Cd^2+^ and Pb^2+^ in live cells.. LG was recently employed [22] to evaluate the HMs content (Cd^2+^, Pb^2+^) in stressed/polluted and not stressed/virtually unpolluted hepatopancreatic cells of terrestrial isopods.

In the current work, the recently developed fluorescent probe (N-(2-(2′-hydroxy-3′-naphthyl)benzoxazol-4-ylmethyl)-N,N-bis(2-aminoethyl)amine), hereinafter referred to as HNBO-DEN [23], is evaluated as potential optical chemosensor for the detection of selected metal ions in solution and in biological environments, both alone and in association with core-shell silica nanoparticles (Si-NPs). The latter were synthesized and conjugated to the HNBO-DEN dye (HNBO-DEN-NPs) with the goal of evaluating the potential use of this fluorescent ensemble for HMs tracing, particularly useful for environmental applications and suitable for FC and CM analyses. Therefore, HNBO-DEN and HNBO-DEN-NPs were tested for Cd^2+^ detection, monitoring the efficiency of the dye and its conjugate in two different models: the human cancer cell line HT-29 and mussel immune cells. The choice of cell models was defined by taking in consideration that some HMs, like cadmium and arsenic, are studied for potential therapeutic benefits in both prostate and hematological malignancies [24, 25] and the possibility to detect their accumulation in cells, could be extremely useful for clinicians. On the contrary, mussels are generally used as marine bioindicators to evaluate chemical pollution [26, 27]. Because they have a metabolism that promotes the accumulation of contaminants [28]. Indeed, when used as food, especially whole, they can transfer these contaminants to humans, posing a potential health risk: this propelled us to employ intestinal HT-29 cells, since they represent a model to investigate diverse pollutant families, including heavy metals, pesticides, persistent organic pollutants, artificial sweeteners, nanomaterials, and other food additives [29–31].

The conjugation of the HNBO-DEN probe to the NF_R_700 NPs did not compromise the ability to detect cadmium in the different models studied but also conferred some advantages: (1) the ability to evaluate the internalization of the NF_R_700 NPs (and the linked HNBO-DEN dye); (2) the possibility to visualize metabolically viable cells due to nanoparticle (NP) uptake (an energy-dependent, active process). This research highlights that both the formulations can be employed as cadmium tracers.

## Materials and methods

### Chemicals

NaF (J.T. Baker-Fisher scientific, Phillipsburg, NJ, USA); SrCl2 × 6H2O (J.T. Baker-Fisher scientific, Phillipsburg, NJ, USA); H3BO3 (J.T. Baker-Fisher scientific, Phillipsburg, NJ, USA); KBr (J.T. Baker-Fisher scientific, Phillipsburg, NJ, USA); KCl (J.T. Baker-Fisher scientific, Phillipsburg, NJ, USA); CaCl2 × 2 H2O (J.T. Baker-Fisher scientific, Phillipsburg, NJ, USA); Na2SO4 (J.T. Baker-Fisher scientific, Phillipsburg, NJ, USA); MgCl2 × 6 H2O (J.T. Baker-Fisher scientific, Phillipsburg, NJ, USA); nahco3 (J.T. Baker-Fisher scientific, Phillipsburg, NJ, USA); NaCl (J.T. Baker-Fisher scientific, Phillipsburg, NJ, USA); CdCl_2_.xH_2_O (Sigma-Aldrich, St. Louis, MO, USA); methyl trimethoxy silane (MTMS) (Merck Millipore, Burlington, MA, USA); cyanine 5.5–NHS (Lumiprobe, Westminster, MD, USA); Proclin ™950 (Merck Millipore, Burlington, MA, USA); sodium carbonate (Merck Millipore, Burlington, MA, USA); sodium hydrogen carbonate (Merck Millipore, Burlington, MA, USA); 2-(n-morpholino) ethanesulfonic acid (MES) sodium salt (Merck Millipore, Burlington, MA, USA); dimethyl sulphoxide (DMSO) anhydrous (Merck Millipore, Burlington, MA, USA); ethylenediaminetetraacetic acid (EDTA) (Merck Millipore, Burlington, MA, USA); trinitrobenzenesulfonic acid (TNBSA) (Merck Millipore, Burlington, MA, USA); edc n-(3-dimethylaminopropyl)-n′-ethylcarbodiimide (Merck Millipore, Burlington, MA, USA); n-hydroxysuccinimide (NHS) (Merck Millipore, Burlington, MA, USA); di-sodium hydrogen phosphate (Merck Millipore, Burlington, MA, USA); sodium dihydrogen phosphate monohydrate (Merck Millipore, Burlington, Massachusetts); sodium chloride (Merck Millipore, Burlington, MA, USA); methyl trimethoxy silane (MTMS) (Merck Millipore, Burlington, MA, USA).

### Resuming chemical

#### Synthesis of HNBO-DEN

HNBO-DEN was synthesized as previously reported [23]. Briefly, the MOM-protected and brominated HNBO was coupled with N,N-bis(2-phthalimidoethyl)amine in DMF in the presence of a base. The product was then deprotected on both amine functions and OH naphthol group in two steps (using hydrazine in EtOH and p-toluensolfonic acid in MeOH, respectively), to give HNBO-DEN, which was finally purified as hydrochloride salt.

#### NPs synthesis

Brij58 (surfactant), dyes and PEG, both functionalized with a triethoxysilane group (fluo-Sil and PEG-Sil), are weighed in a flask and dissolved with the minimal amount of dichloromethane. The solvent is removed and then the solid is dissolved in water + 1-butanol (15 µL every 1 ml of water). The solution is maintained under stirring and placed at 40 °C. At this point MTMS (methyl trimethoxy silane) is added (final concentration of 100 mM). After 10 min a 0.3 M DMSO solution of PEG functionalized with an amino group and a triethoxy silane group (NH2-PEG-Sil) is added, final concentration 1.75 mM. After 10 min a 2.8% v/v aqueous ammonia solution is added (15ul every 1 ml of solution). The mixture is left under stirring at 40 °C for 2 days. After 48 h of biobeads SM2- adsorbents (Biorad 1523920) are added (0.012 g every mg of Brij58) at room temperature. After stirring for 3 h, the surfactant is removed. The solution is then filtered to remove the beads and dialyzed for at least 24 h and 3 changes of water with 12–14 kDa MWCO dialysis membrane. NPs are filtered 2 times with 0.22-mircon filters, centrifuged at 8000 rcf for 30 min and filtered again with a 0.1-micron filter. To synthesize Si-NPs, the technique adopted in the work of Pellegrino et al*.* [32] was used. The synthesis starts with the mixing of a surfactant with water, *n*-butanol and polyethylene glycol functionalized with a triethoxysilane group (PEG-Sil). In these conditions, the surfactant creates micelles, hence the process is known as micelle-assisted method (see Supplementary Materials).

### HNBO-DEN and NF_R_700 silica nanoparticle conjugation

For the conjugation procedure between NF_R_700 and HNBO-DEN ([NH2] = 610 µM), 4 mL (2.44·10^3^ nmol = 1 eq of amine groups) of NPs was dialyzed, under stirring, at 4 °C to remove ProClin™ (a preservative purchased from Merck Millipore, added at the end of the synthetic procedure) and to change the buffer in 2 L of Na2CO3/NaHCO3 pH 9.5 a 50 kDa dialysis membrane was chosen. Buffer was renewed after 6 h and then NPs were left overnight in dialysis. The following day, 3.840 mL of NF_R_700 (slightly concentrated) was recovered into a 10 mL glass vial for reacting amine groups with succinic anhydride and converting them to carboxylate. A 40 mg/mL succinic anhydride solution was prepared by dissolving 39.8 mg of succinic anhydride in 995 µL of DMSO. 440 µL (200 eq = 461 µmol) of this solution were added to the vial with the NPs. The mixture was allowed to react at a controlled temperature of 40 °C for 1 h under magnetic stirring. After 1 h the mixture was left to cool down to room temperature, then the dialysis procedure was repeated to change the buffer (MES/EDTA pH 5.5) before the second step of the conjugation. 5.196 mL was recovered the next day. The estimated quantity of carboxylic groups [-COOH] was 2.44·10^3^ nmol at 470 µM concentration (assuming 100% reaction yield). The reaction yield was assessed using TNBSA (Trinitrobenzensulfonic acid) colorimetric method. TNBSA reacts with amines generating a chromogenic compound that absorbs at 335 nm. Assessing unreacted primary amine, reaction yield and [-COOH] can be obtained. The [-NH_2_] was assessed with the following procedure (see Supplementary Materials). The reaction yield is estimated to be greater than 98%, therefore in the following stoichiometric calculation a 100% yield has been assumed in the conversion of -NH_2_ to -COOH. Considering that the [-COOH] is 470 µM and the remaining volume is 4.996 mL, 2.35·10^3^ nmol of -COOH = 1 equivalent. Ultrafiltration was then performed with a 50 kDa PES (polyethersulfone) filter to bring the NPs to 4.0 mL (final concentration [-COOH] = 587 µM). A solution of EDC (1-Ethyl-3-(3-dimethylaminopropyl)carbodiimide) was prepared by adding 20 µL of EDC to 180 µL of DMSO. A 20 mg/mL aqueous solution of NHS (N-Hydroxysuccinimide) was prepared. The two solutions (4 eq) were added to the tube containing the NPs and reacted for 1.5 h at room temperature. In the meantime, a solution of HNBO-DEN in water was prepared by dissolving 3.7 mg in 500 µL (3.5 eq). After 1.5 h the solution of HNBO-DEN was added. To bring the pH to 7.5, 500 µL of 100 mM Na2CO3/NaHCO3 pH 9.5 buffer was added. The mixture was left reacting for 1.5 h at room temperature, then the reaction mixture was dialyzed against PBS (phosphate buffer saline) 10 mM NAPI/150 mM NaCl pH 7.35 at 4 °C in the dark.

**Fig. 1:**
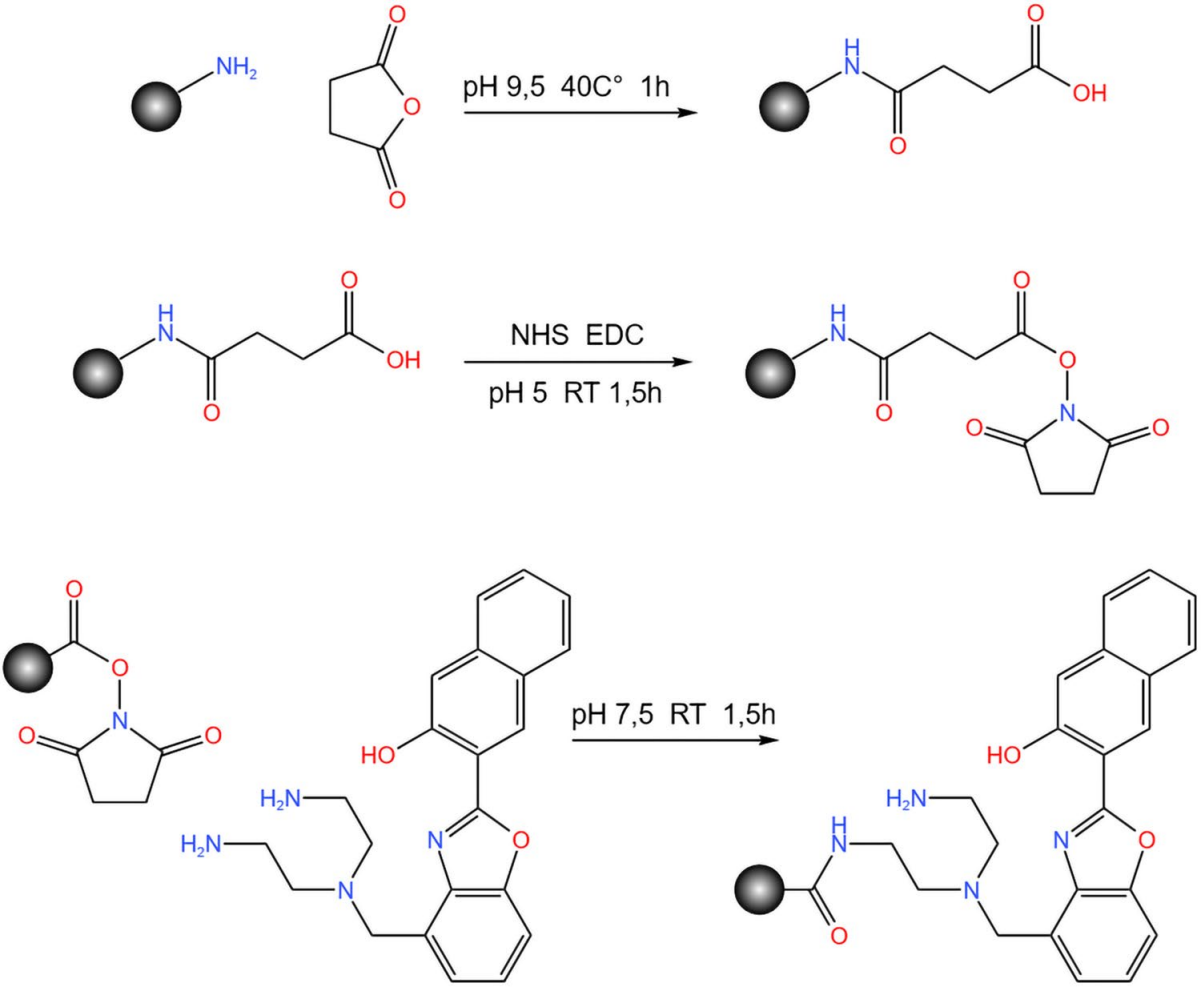
Schematization of preparative and conjugation chemical reactions of NPs with HNBO-DEN

All preparation and conjugation reactions of NPs with HNBO-DEN are shown in Fig. 1.

### Cell culture conditions and treatments

HT-29 is a human colorectal adenocarcinoma cell line (kindly provided by AcZon srl, original source: Merck). The cells were cultured in RPMI 1640 Medium + 1% L-glutamine (Euroclone SPA, Pereo (MI), Italy) supplemented with 10% Heat-Inactivated Foetal Bovine Serum (FBS; Euroclone SPA, Pereo (MI), Italy), and 1% penicillin/streptomycin (Biosigma S.p.A. Cona (VE), Italy) at 37 °C in humidified air with 5% CO2. HT-29 cells were seeded in 6-well plates at a density of 1.5 × 10^5^ cells per well and exposed to different concentrations (50 µM, 100 µM and 150 µM) of a solution of CdCl_2_ (10 mM) and incubated for 45 min and 24 h. For the control, the cells were incubated with a complete medium only.

### *Mytilus galloprovincialis* Hemolymph extraction and treatments

Specimens of the bivalve molluscs *Mytilus galloprovincialis* were purchased from a local fishing company (MARR Spa Rimini, Italy). Analyses were carried out on individuals of 4–5 cm size. After collection, animals (30 mussels) were taken immediately to the laboratory where they were kept in an aquarium for 24 h in static tanks containing 36 ppt artificial sea water (ASW) (1 L mussel^−1^) at 16 °C; sea water was changed daily. Hemolymph was sampled from the posterior adductor muscle using a sterile 1 ml syringe with an 18 G1/2″ needle. With the needle removed, hemolymph was filtered through sterile gauze and pooled in 50 ml centrifuge tubes at 18 °C. Hemolymph samples from 8 to 10 mussels were pooled and utilized for subsequent analyses. CdCl_2_ from a CdCl_2_ stock solution was suitably diluted in filtered ASW and added to the hemolymph to reach the desired concentration. Hemolymph was exposed for 30 min and 1 h to either CdCl_2_ (1 mM, 1 μM, and 1 nM). A parallel group of control (untreated) hemolymph was kept in filtered ASW.

### Flow cytometric (FC) analyses

FC experiments were conducted using a FACSCanto II flow cytometer (BD Biosciences, San Jose, CA, USA) equipped with an argon laser (488 nm, blue excitation), a helium-neon laser (633 nm, red excitation), and a solid-state diode laser (405 nm, violet excitation) and NovoCyte® 3000 flow cytometer (ACEA Biosciences, San Diego, CA, USA), equipped with three lasers (Violet Ex 405 nm, Blue Ex 488 nm and Red Ex 640 nm). Data acquisition and analysis were performed using FACSDiva™ software (BD Biosciences, San Jose, CA, USA) and Kaluza software (Beckman Coulter, Brea, CA, USA). At least 10,000 events were acquired for each sample.

The new fluorescent probe (HNBO-DEN) was dissolved in DMSO at a concentration of 15 mM for detecting the presence of Cd^2+^ and it was used at 500 nM final concentration for 20 min at RT, showing good staining performance. The NF_R_700-HNBO-DEN was employed as fluorescent probe at the same concentration of HNBO-DEN. Cell viability was evaluated using propidium iodide (PI; Sigma-Aldrich) and 7-AAD (Beckman Coulter, Brea, CA, USA) staining. Cells were incubated in the dark for 10 min with PI (1 mg/mL) and/or 7-AAD. The percentage of PI- or 7-AAD-positive cells was measured as an indicator of overall cellular damage.

### Confocal microscopy

Confocal microscopy analyses were applied by a Leica TCS SP5 II confocal microscope (Leica Microsystem, Germany) with 488, 543, and 633 nm illuminations and oil-immersed objectives. For confocal live imaging, cells were grown on MatTek glass bottom chambers (MatTek Corporation, Bratislava, Slovak Republic). The images were further processed and analyzed in ImageJ software (NIH, Bethesda, MD, USA).

### DLS analysis

The samples were analyzed with Nanotrac Flex DLS instrument (Microtac Inc, Montgomeryville and York, Pennsylvania, PA, USA) equipped with 780 nm laser. No dilution was needed. The results shown are the average of 4 measures, set time for each scan was 60 s.

### Transmission electron microscopy (TEM) analysis

Conjugated and unconjugated NP suspensions for TEM were diluted at a ratio of 1:100 with Milli-Q® water.

10 µL of the diluted solution was placed on carbon/formvar supp. films on 200 mesh copper grids (Ted Pella inc, Redding, CA, USA). After 5 min, the drop was blotted and the grid allowed to dry at room temperature overnight.

The sections were examined using a HT7800 transmission electron microscope (HITACHI, Ibaraki, Japan) at 100 kV.

### Statistical analysis

Data are shown as mean (or percentage, as indicated) ± standard deviation (SD) of at least three independent experiments. To make a comparison among experimental groups comprising more than two elements, a statistical approach, specifically Analysis of Variance (ANOVA), was employed, provided that the normality assumption was held. Subsequently, one- and two-way ANOVA were followed by a post-hoc Bonferroni test. Statistical significance was attributed to p-values less than 0.05. Bonferroni’s multiple comparison test revealed statistically significant: *p < 0.05, **p < 0.01, ***p < 0.001, ****p < 0.0001. All statistical analyses were performed using GraphPad Prism 9.0.0 (GraphPad software, San Diego, CA, USA).

## Results and discussion

### Synthesis of HNBO-DEN and HNBO-DEN-NPs

HNBO-DEN is a recently developed fluorescent probe based on HNBO (2-(2-hydroxy-3-naphthyl)−4-methylbenzoxazole), in which a fluorophore moiety is bound to a N,N-bis(2-aminoethyl)amine receptor unit. HNBO-DEN is a fluorescent molecule possibly featuring an excited state intramolecular proton transfer (ESIPT) process that has already shown the ability to bind different metal ions (such as Mg^2+^, Cd^2+^, and Zn^2+^) with different selectivities in different solvents. In particular, it was selective for Mg^2+^ in DMSO and ACN compared to alkali, alkaline-earth, and transition metal ions, while it only responded to Zn^2+^ and Cd^2+^ in water and alcohols [23]. The fluorescence response to Cd^2+^ was found to be linear up to 3.42 ppm (Figure S1). NF_R_700 NPs were chosen for the conjugation to HNBO-DEN. Such NPs were doped with cyanine 5.5 dye (epsilon 277,000 M^−1^ cm^−1^ measured in the particles), and their absorption/emission maxima were 686 and 712 nm, respectively. Their fluorescence quantum yield in water is 0.19, relative to cyanine 5.5 in ethanol as a reference. The mean diameter measured with DLS was around 9 nm, while the estimated concentration of reactive amine groups on the shell was [-NH2] = 610 µM. NF_R_700 resulted in the best choice for conjugation to HNBO-DEN, since their excitation wavelength (633 nm) does not interfere with that of the dye, allowing for the selective excitation of HNBO-DEN (405 nm). Moreover, even if the emission spectra do not overlap, compensation due to spillover is not needed.

### Characterization of NPs and HNBO-DEN-NPs

**Fig. 2:**
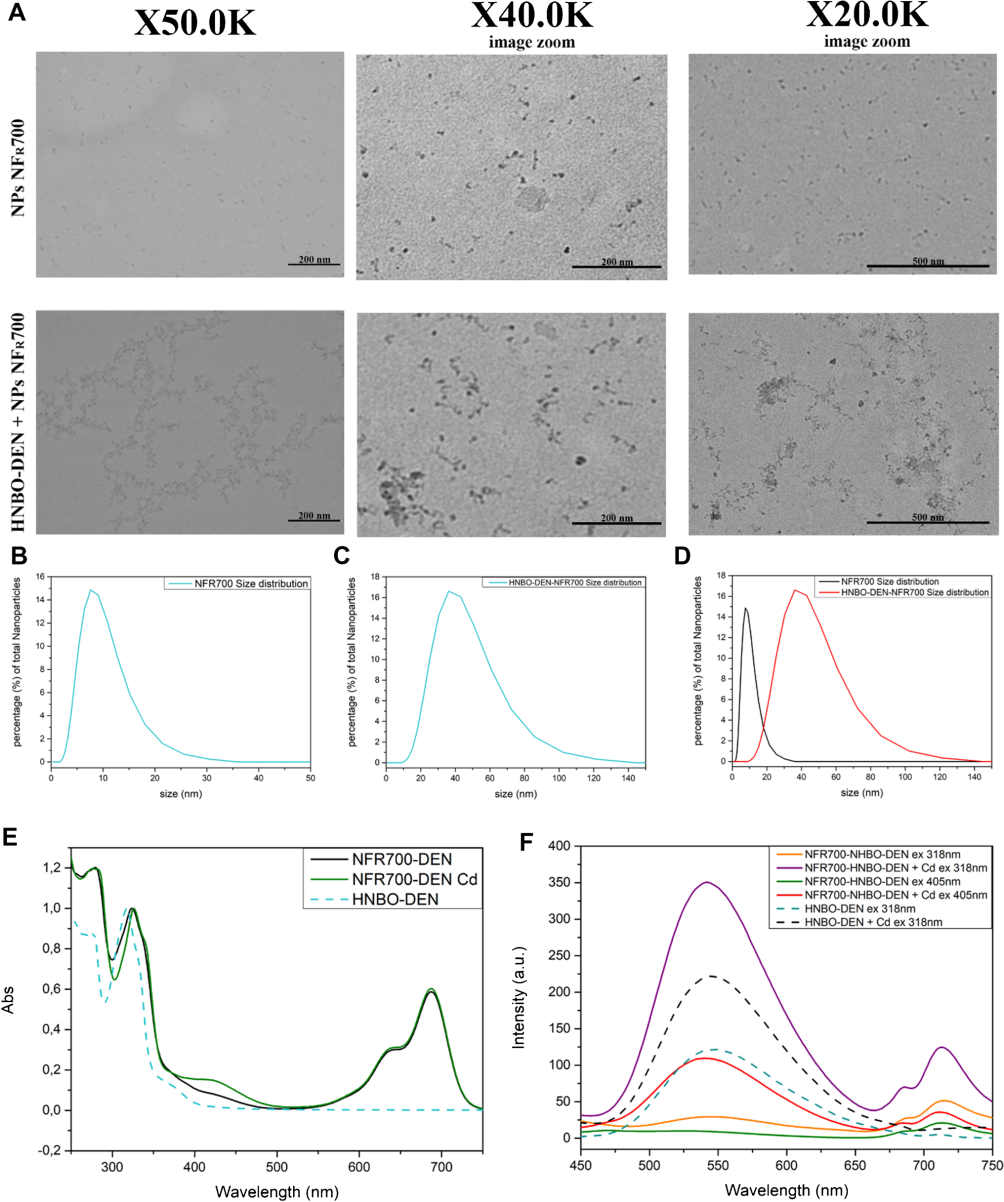
Morphological and spectroscopic characterization of NF_R_700 and the HNBO-DEN-NF_R_700 construct. **A** TEM micrographs of NF_R_700 and the HNBO-DEN-NF_R_700 construct at × 20.0 K, × 40.0 K, and × 50.0 K magnifications. **B**–**D** Size distribution of NF_R_700 and the HNBO-DEN-NF_R_700 construct obtained by DLS measurements. The addition of organic layers (specifically COOH groups) to the construct increases the overall hydrodynamic diameter while the spherical morphology of the silica core is maintained, as observed in TEM. A higher degree of aggregation is visible in TEM, particularly with high dye loading. **E** Absorption spectra of HNBO-DEN-NF_R_700 (black), HNBO-DEN-NF_R_700 with Cd^2+^ (green) and free HNBO-DEN (light blue). **F** Emission spectra of HNBO-DEN-NF_R_700 (orange and green), HNBO-DEN-NF_R_700 with 1 equiv. Cd^2+^ (purple and red), free HNBO-DEN (blue) and HNBO-DEN with 1 equiv. Cd^2+^ (black). [HNBO-DEN]: 1.2 × 10^−5^ M; emission intensity is normalized at 1 mM HNBO-DEN

The analyses were mainly conducted by TEM (Fig. 2A) and DLS (Fig. 2B, C, and D).

The DLS results are consistent with the TEM observations. Both in the case of the NP sample (NF_R_700) and the NP conjugate with HNBO-DEN, the analysis was performed on particles resuspended in water after 15 min of sonication, i.e., the same procedure followed to prepare the samples for dispersion in the growth medium (Fig. 2). The NPs show a size ranging from 5 to 15 nm with an average distribution of around 7 nm. These sizes are similar to those proposed by previous studies on the same type of particles [33–36].

The NPs conjugated with HNBO-DEN showed a size ranging from 20 to 70 nm with an average distribution of around 35 nm.

The emissions of NF_R_700 nanoparticles perfectly mimic those of commercial dyes used for flow cytometry and microscopy (Cy5.5). As is known, other dyes can be used efficiently due to solubility in organic solvents and the presence of a trialkoxysilane group.

Absorption spectra (Fig. 2E) clearly show the HNBO-DEN successfully conjugated to NF_R_700; indeed, the HNBO-DEN characteristic band around 325 nm appears, even if it is slightly red-shifted. Since the absorption maximum of HNBO-DEN shifts to longer wavelengths as the solvent polarity decreases (317 nm in ACN, 320 nm in DMSO; see material and method section), this suggests that the local environment is less polar than water. This indicates that HNBO-DEN is not freely dissolved but is likely covalently bound to the PEG shell. As further evidence, the HNBO-DEN peak remains detectable even after dialysis, which is consistent with this interpretation.

From spectrophotometric data the estimated reaction yield is 96% (2.25·103 nmol of HNBO-DEN conjugated) according to the following formula:

$$[HNBO-DEN]=\frac{{A}_{318nm}-{\mathrm{A}}_{687nm}\frac{{\epsilon}_{318nm\;Cy5.5}}{{\epsilon}_{687nm\;Cy5.5}}}{{\epsilon}_{318nm\;Den}}$$

The absorption coefficient estimated in previous work of HNBO-DEN at maximum absorption is used [23].

Adding Cd^2+^ to a solution of HNBO-DEN-NF_R_700 slightly changed the shape of the band in the UV region, nevertheless a band at around 425 nm appeared (green line in Fig. 2F). This band is ascribable to the deprotonation of the naphthol moiety of the HNBO fluorophore, which is due to the coordination of the metal ion and is responsible for the observed increase of emission [23].

This behavior is also found for the free ligand upon addition of the same metal ions, as reported by Paderni et al. [23] and demonstrates that the conjugated ligand HNBO-DEN did not lose the chelating capability even if one primary amine was engaged in amide bond formation (Fig. 1).

Notably, the presence of the absorption band at 425 nm produces a remarkably brighter emission (see also data in Table 1) compared to that at 326 nm. Emission spectrum (Fig. 2F) confirms what was shown in Paderni et al. 2023 [23]. The HNBO-DEN construct (purple line), or free (light blue line) ligand is turned OFF, no significant emission is registered. Upon addition of Cd^2+^ the fluorescence emission is turned ON (green lines and red line) and the emission intensity at 540–548 nm increased (Fig. 2F). Thus, HNBO-DEN-NF_R_700 could be considered as a suitable fluorescent chemosensor for sensing Cd^2+^. Moreover, since HNBO-DEN is not highly fluorescent in the absence of some bivalent metal ions, NPs provide a tool for tracking the conjugate in a biological environment. The transmittance normalized emission intensity, calculated as:

$$\frac{{I}_{em}}{\left(1-{10}^{-A}\right)}$$

allowed for comparing the emission values of different solutions, even if their absorbances were different, indeed this is equivalent to say that all the solutions were prepared with the same transmittance and hence the same absorbance. Since the absorbance of all solutions is the same, the more the transmittance normalized intensity the more efficient is the fluorophore. From the data shown in Table 1, it is evident that the addition of 1 equivalent of Cd^2+^ increases the normalized emission intensity and so the efficiency of HNBO-DEN, even conjugated to the NF_R_700 NPs.

**Table 1:** Transmittance normalized emission intensity of different aqueous solutions

	Transmittance normalized emission intensity
DEN	2.9
DEN + 1 eq Cd^2+^	9.9
HNBO-DEN-NF_R_700 ex 318 nm	1.6
HNBO-DEN-NF_R_700 + 1 eq Cd^2+^ ex 318 nm	11.3
HNBO-DEN-NF_R_700 ex 405 nm	4.7
HNBO-DEN-NF_R_700 + 1 eq Cd^2+^ ex 405 nm	16.3

### HNBO-DEN cell staining

#### Analysing cadmium content in intestinal HT-29 cells: does HNBO-DEN perform well?

First, we performed several evaluations of possible dye cytotoxic concentrations: with HT29 cells as experimental model. At the highest dye concentrations employed (50 and 100 micromolar), HNBO-DEN is toxic for the cells (Figure S2). However, these are 100- and 200-fold higher than the final working solutions, respectively, and the concentration revealing minimal cytotoxicity is three-fold higher than that one selected for final evaluations (Figure S2) Furthermore, as often observed with similar probes for evaluating cell functions [37, 38] the highest dye concentrations are harmful for the cells.

As channels to collect HNBO-DEN fluorescence (considering the emission features of the large dye emission spectrum, a characteristic yet widely reported [39]), we tested both AmCyan and FITC fluorescences, considering that AmCyan’s emission wavelength peaks around 489–498 nm, whereas FITC (Fluorescein Isothiocyanate) corresponds to an emission wavelength peaking around 519–525 nm. AmCyan MFI (mean fluorescence intensity) data are shown in Figure S3 and, although MFI trend values are similar to those from FITC, significance is almost absent, probably due to the greater contribution of cell self-fluorescence in this spectral interval.

As part of our pre-evaluation, we highlighted the advantages of utilizing this class of dyes in live, functional cells, as opposed to the destructive approaches outlined in Supplementary Figure S4, A. Comparative analysis with the commercially available Leadmium™ Green dye was conducted using the homogeneous HT-29 model. Assessment of the MFI ratio (treated/untreated) demonstrated that HNBO-DEN (Figure S4, B) and Leadmium™ Green (Figure S4, C) exhibit comparable performance, confirming assay sensitivity (Figure S4, D).

To better assess the ability of the HNBO-DEN dye to detect intracellular HMs content, particularly cadmium, HT-29 intestinal cells were exposed to different concentrations of CdCl_2_ (50 µM, 100 µM, 150 µM) and different times (45 min and 24 h).

**Fig. 3:**
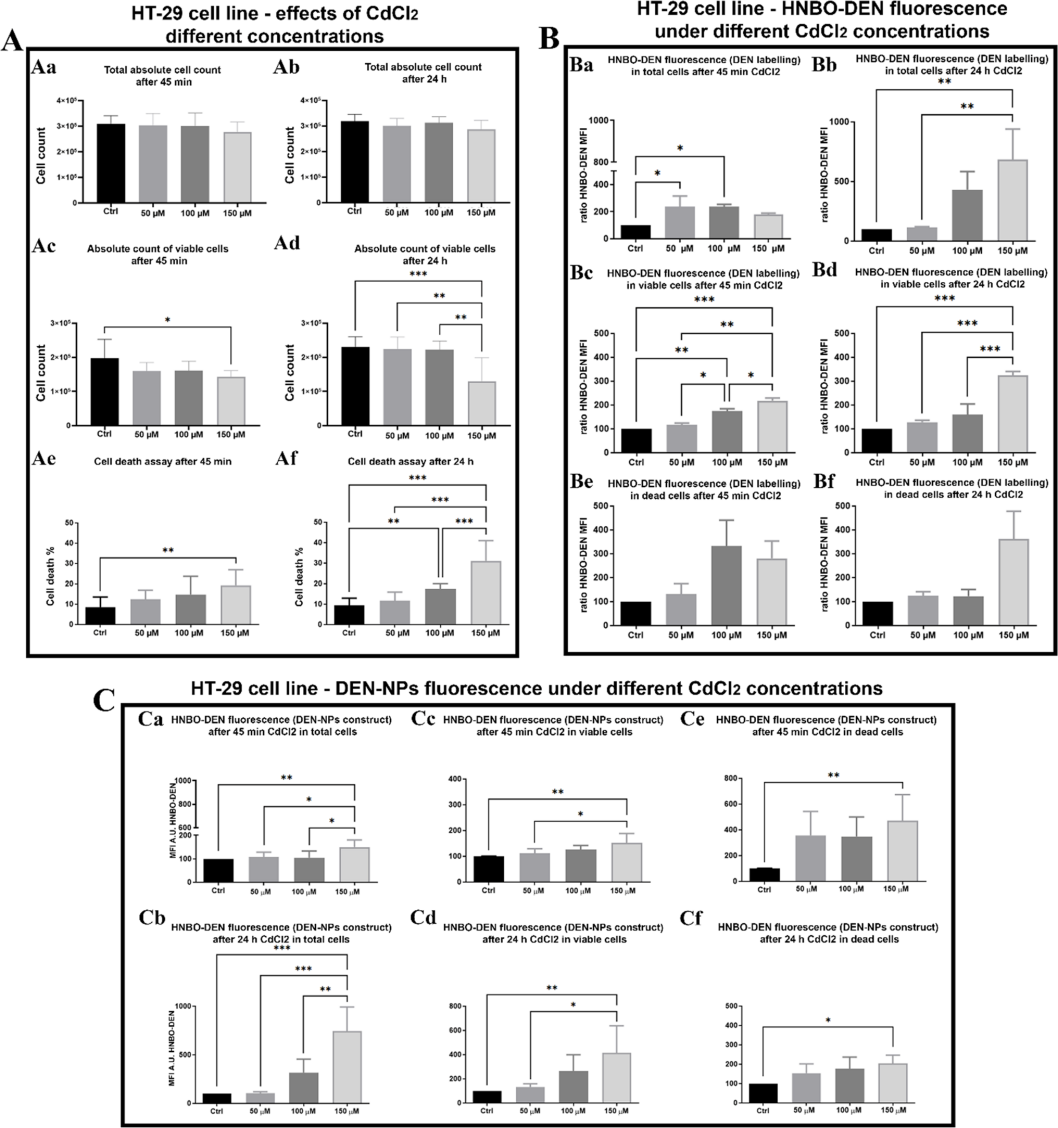
HT-29 cell line—effect of CdCl_2_ at different concentrations and HNBO-DEN/DEN-NPs fluorescence in CdCl_2_ conditioned cells.** A** HT-29 cell line—effects of CdCl_2_ at different concentrations. Statistical histograms representing the total absolute cell count at two time points: (Aa) after 45 min and (Ab) after 24 h of treatment with Control (Ctrl) or CdCl_2_ (50, 100, and 150 uM). The absolute count of viable cells is shown for (Ac) 45 min and (Ad) 24 h. Cell death assay results are presented as percentages for (Ae) 45 min and (Af) 24 h. **B** HT-29 cell line—HNBO-DEN fluorescence under different CdCl_2_ concentrations. Histograms showing the ratio of HNBO-DEN Mean Fluorescence Intensity (MFI) in total cells after (Ba) 45 min and (Bb) 24 h; in viable cells after (Bc) 45 min and (Bd) 24 h; and in dead cells after (Be) 45 min and (Bf) 24 h. This evaluation shows that after 45 min a significant increase in HNBO-DEN fluorescence occurs at 50 uM and 100 uM CdCl2. At 24 h, MFI in viable cells (Bd) reflects a dose-dependent sustained accumulation, while signal in dead cells (Bf) is inconsistent due to membrane leakage. **C** HT-29 cell line—HNBO-DEN-NF_R_700 NP fluorescence CdCl_2_ conditioned cells. Statistical histograms of HNBO-DEN MFI using the DEN-NPs construct in total cells at (Ca) 45 min and (Cb) 24 h; in viable cells at (Cc) 45 min and (Cd) 24 h; and in dead cells at (Ce) 45 min and (Cf) 24 h. This evaluation confirms the nanoparticle’s ability to detect the fold of increase of intracellular cadmium, highlighting a rapid initial entry followed by a slower, sustained accumulation phase up to 24 h (Cb, Cd, Cf). Data are expressed as mean ± SD. One-way ANOVA with Bonferroni multiple comparison revealed: *p < 0.05, **p < 0.01, ***p < 0.001

Firstly, the possible cell loss induced by Cd^2+^ was evaluated through cell count assessment (Fig. 3Aa and Ab) and cell death detection (Fig. 3Ac and Ad) [40, 41]. However, after a short exposure of 45 min, a negligible total cell reduction can be observed at concentrations of 50 µM and 100 µM (Fig. 3A); indeed, cell death percentages were found also mild at 150 µM concentration (Fig. 3Ae), suggesting that this tumoral cell line is not particularly sensitive to Cd^2+^ toxicity in short time exposure. Such findings are in agreement with Naji and coworkers [42] showing that Cd^2+^ at low doses can even potentiate the migratory capacity of HT-29 cells. After 24 h, the reduction in total cell count is still not significant (Fig. 3B), but with evidence of relevant cell death in 150 µM CdCl_2_-treated cells (Fig. 3Af), in agreement with several previously reported works [43–47]. More in depth, Fig. 3Ac and Ad highlight viable HT-29 cell counts after 45 min and 24 h, respectively. From the integration of data in panel A (Fig. 3), a scenario of a slow, controlled cell death (compatible with apoptosis) is depicted, in agreement with Hajrezaie and coworkers [48]. Detection of viable and dead cells (still present in samples) allowed to elucidate and biologically interpret data on HNBO-DEN fluorescence after both 45 min and 24 h CdCl_2_ treatment (Panel B of Fig. 3).

The HNBO-DEN ability to reveal Cd^2+^ content on the HT-29 cell line was investigated to determine whether the dye can reveal the presence and the fold of increase of cadmium inside the cells also after 24 h. After a 45 min brief exposure, a slight but significant increase in HNBO-DEN fluorescence occurs in total cells treated by 50 µM and 100 µM CdCl_2_, this mild increase appears not significant in cells treated by 150 µM. Of note, if the analysis is limited to viable cells, HNBO-DEN MFI appear more consistent with cadmium doses (Fig. 3Bc vs 3Bd), whereas dye fluorescence in dead cells does not follow the same trend, similarly after 24 h CdCl_2_ treatment (Fig. 3Bd vs Bf). These effects can be explained by the following processes: (1) during cell death an uncontrolled leakage due to membrane rupture, can lead to releasing each cell content, including pollutants; (2) cadmium penetrates cells through a rapid passive transport process occurring in seconds to minutes, followed by a slower, sustained accumulation phase, representing the most damaging cell event. HT-29 findings during time highlight this sustained entry pattern, with an initial rapid phase and a slower, sustained Cd^2+^ accumulation within the cell that continues for minutes to 24 h (Fig. 3Bb, Bd, Bf).

#### HNBO-DEN-NPs on cell culture

Being HNBO-DEN-NF_R_700, a novel nanochrome that may exhibit a different behavior from that of the free form (HNBO-DEN dye), it was also tested on HT-29 cells. At shorter time points (45 min), the HNBO-DEN-NPs showed MFI values significantly increased only for the 150 µM concentration (Fig. 3Ca–e). At the end of the time course (24 h), HNBO-DEN-NF_R_700 highlighted brighter fluorescence, with an increase proportional to cadmium doses. It is noteworthy that only the treatment with CdCl_2_ at 150 µM resulted in a significant increase compared to all other conditions (Fig. 3Cb–f).

Viable/dead cells separation confirms the same evidence emerged by HNBO-DEN labelling, although in dead cells a significant MFI increase is detected after both 45 min and 24 h treatments. Indeed, a gain in the fold of increase in MFI values is appreciable in Fig. 3C (in respect of Fig. 3B), suggesting that HNBO-DEN-NP construct is particularly effective in detecting intracellular cadmium bioaccumulation in primary organelles, mainly represented by mitochondria [49], endoplasmic reticulum [50], and lysosomes [51]. Such differential behavior for HNBO-DEN and HNBO-DEN-NP construct can be sustained by confocal images of Figure S5, highlighting not only confirmation of FC quantitative data, but also a diffuse green fluorescence for the dye alone and a punctate green fluorescence for HNBO-DEN-NPs. Such analyses support the hypothesis thata specific fluorescence in intracellular cadmium accumulation sites.

Finally, the monitoring of the intrinsic NP fluorescence in HT-29 cells at both times investigated (Figure S6) showed a similar fluorescence trend, able to confirm the amount of internalized NPs and, consequently, of HNBO-DEN (Fig. 3C).

#### HNBO-DEN on hemolymph cells of *Mytilus galloprovincialis*

To assess the ability of the HNBO-DEN dye to detect intracellular Cd^2+^ content in environmental bioindicators, hemolymph cells from *Mytilus galloprovincialis* were employed*.* For the study, hemocytes from healthy individuals were exposed to different concentrations of CdCl_2_ (1 mM, 1 µM, 1 nM) at different times (30 min and 1 h). Cadmium concentration of 1 nM (nM) is approximately consistent with background levels in coastal seawaters and this is the lowest concentration we adopted. Considering values for seawater [52] and fish products [53] the concentrations of CdCl_2_ employed in our in vitro system seem to cover the different ecologic scenarios. Longer incubation times were excluded, as both hyalinocytes (small cells) and granulocytes (large cells) tend to suffer excessive death rates in similar ecotoxicological studies [54].

**Fig. 4:**
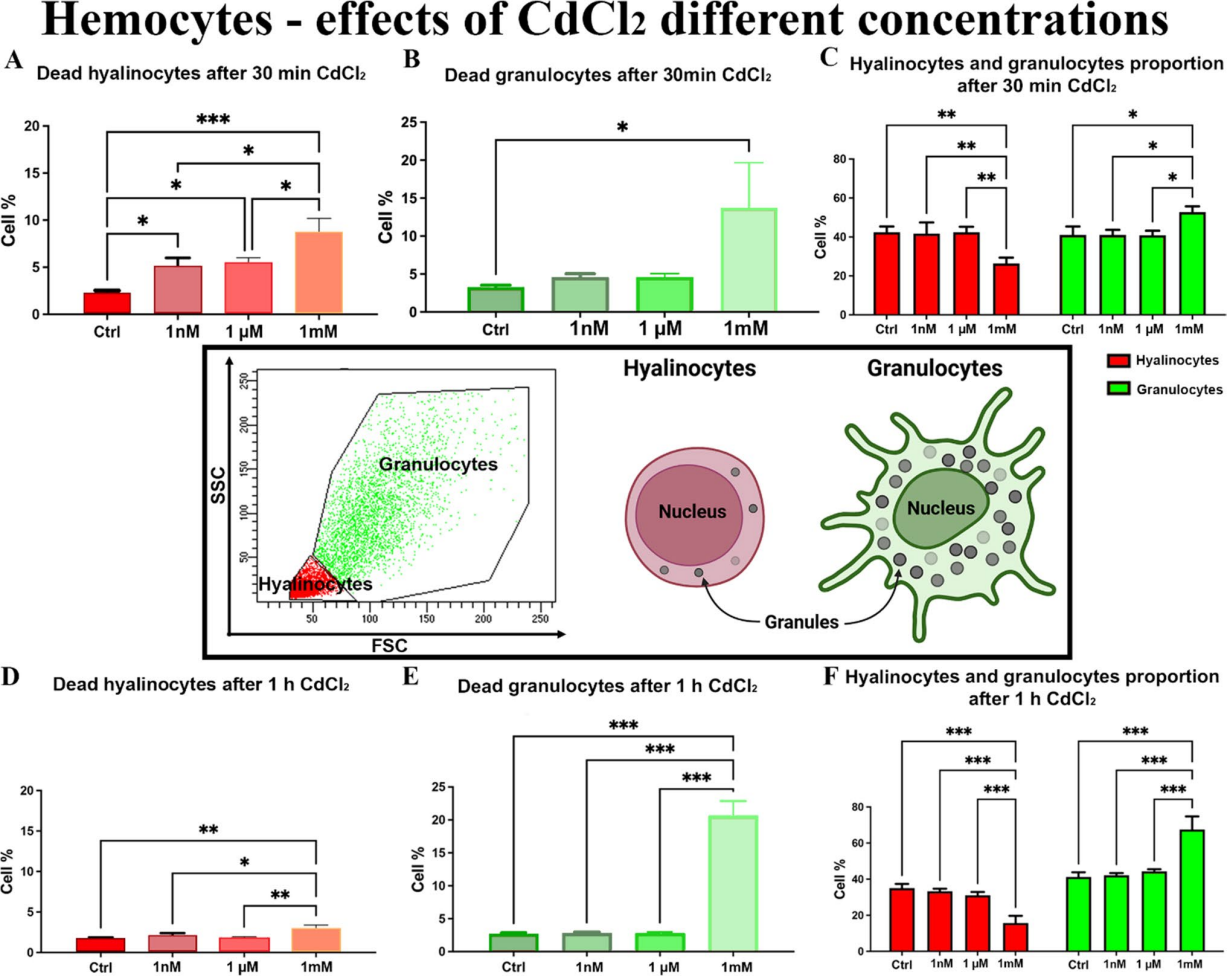
CdCl_2_ different concentrations: effects on hemocyte dead cells and homeostasis.** A** Statistical histograms of percentage of dead cells in hyalinocytes at 30 min for control (CTRL) and treated cells with CdCl_2_ at different concentrations (1 nM, 1 µM, and 1 mM). **B** Statistical histograms of percentage of dead cells in granulocytes at 30 min for control (CTRL) and treated cells with CdCl_2_ at different concentrations (1 nM, 1 µM, and 1 mM). **C** Statistical histograms of percentage of hyalinocytes and granulocytes at 30 min for control (CTRL) and treated cells with CdCl_2_ at different concentrations (1 nM, 1 µM, and 1 mM). In the middle: black square presents a representative flow cytometry dot plot illustrating the analysis of the hemocyte population. Dot plot of forward scatter (FSC) vs side scatter (SSC) is employed to discriminate two primary subpopulations: granulocytes and hyalinocytes. Adjacent a scheme of the morphologies of both cell types, highlighting that hyalinocytes (or agranulocytes), contain few or no granules within the cytoplasm and a higher N/C (nucleus-to-cytoplasm) ratio and exhibit lower phagocytic activity than granulocytes. **D** Statistical histograms of percentage of dead cells in hemocytes at 1 h for control (CTRL) and treated cells with CdCl_2_ at different concentrations (1 nM, 1 µM, and 1 mM). **E** Statistical histograms of percentage of dead cells in granulocytes at 1 h for control (CTRL) and treated cells with CdCl_2_ at different concentrations (1 nM, 1 µM, and 1 mM). **F** Statistical histograms of percentage of hyalinocytes and granulocytes at 1 h for control (CTRL) and treated cells with CdCl_2_ at different concentrations (1 nM, 1 µM, and 1 mM). One and Two-way ANOVA with Bonferroni’s multiple comparison revealed: *p < 0.05, **p < 0.01, ***p < 0.001

Firstly, cadmium-induced cell death was assessed in hyalinocytes and granulocytes (Fig. 4). After 30 min, the percentage of dead hyalinocytes appeared to be proportional to the cadmium concentration, nevertheless, at the highest concentration, the percentage of dead cells was mild (10%) (Fig. 4A). In granulocytes, only the highest cadmium concentrations caused a significant cell death rate, reaching 15% of the total cells (Fig. 4B).

The possible alteration in the relative proportion of hyalinocytes and granulocytes was also evaluated, since it represents a signal of a perturbed homeostasis: the treatment with 1 mM CdCl_2_ proved to be harmful to both types of hemocytes and altered their respective relationships (Fig. 4C). To investigate the effect of different concentrations of cadmium after 1 h, the percentage of dead hyalinocytes and granulocytes has been evaluated. Differential hemocyte count (DHC) by FC is able to discriminate between hyalinocytes and granulocytes in each condition and each experimental point, indeed, Ayhan and coworkers [55] found the highest percentages of total granulocytes (both basophilic and eosinophilic) in the pollutant areas, corresponding to our in vitro results. Indeed, only in granulocytes, a significant increase in the rate of dead cells was found (about 25%) (Fig. 4D, E).

The distribution between hyalinocytes and granulocytes was also considered in the cells treated with different increasing concentrations of CdCl_2_ (1 nM, 1 µM and 1 mM) after 1 h; a significant decrease in the percentage of hyalinocytes was registered in 1 mM CdCl_2_ -treated cells appearing counterbalanced by a significant increase in granulocyte percentages (Fig. 4F).

**Fig. 5:**
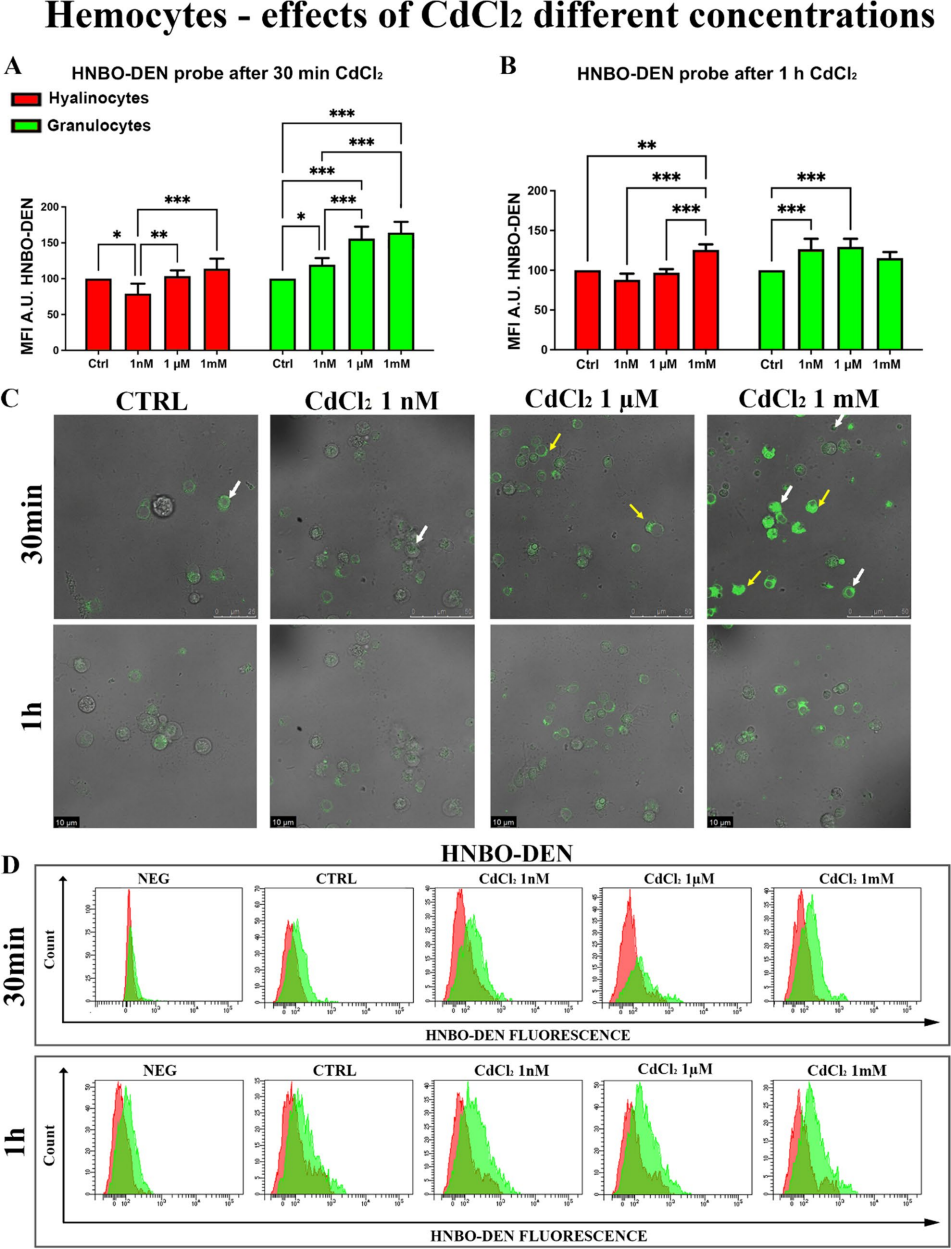
Hemocytes—effects of CdCl_2_ different concentrations. **A** HNBO-DEN probe after 30 min CdCl_2_ exposure. Statistical histograms of HNBO-DEN Mean Fluorescence Intensity (MFI) in hyalinocytes (red bars) and granulocytes (green bars) after 30 min of treatment with control (Ctrl) or CdCl_2_ at different concentrations (1 nM, 1 µM, and 1 mM). Significant increases in fluorescence are observed in both cell types starting from 1 µM. **B **HNBO-DEN probe after 1 h CdCl_2_ exposure. Statistical histograms of HNBO-DEN MFI in hyalinocytes (red bars) and granulocytes (green bars) after 1 h of treatment with control (Ctrl) or CdCl_2_ at different concentrations (1 nM, 1 µM, and 1 mM). The data show sustained fluorescence levels, particularly in granulocytes at 1 µM and 1 mM. **C** Confocal microscopy analysis. Single confocal optical sections of HNBO-DEN (green) in *Mytilus galloprovincialis* hemocytes at 30 min and 1 h for control (Ctrl) and treated cells (1 nM, 1 µM, and 1 mM). White arrows indicate a perinuclear distribution, while yellow arrows highlight a cup-shaped distribution of the HNBO-DEN probe. Scale bars are provided at 10 µM. **D **Flow cytometric analysis. Cytometric histogram overlays of HNBO-DEN fluorescence in hyalinocytes (red) and granulocytes (green) for all conditions (NEG, CTRL, 1 nM, 1 µM, and 1 mM) at both 30 min and 1 h time points. The shift in peaks represents an increase in intracellular cadmium detection. Data are expressed as mean ± SD. Two-way ANOVA with Bonferroni multiple comparison revealed: *p < 0.05, **p < 0.01, ***p < 0.001

Mussel hyalinocytes are sensitive to and can absorb environmental cadmium. However, in Fig. 5A, large granulocytes highlighted they are more sensitive to detrimental effects of cadmium than other hemocyte types: therefore, we considered granulocytes as the best subpopulation to trace the presence of Cd^2+^ within the examined samples. Consistently, HNBO-DEN MFI values (Fig. 5) agreed with the previous evaluations and reported a valuable performance of the dye. The images obtained through CM confirmed FC data, revealing not only the increase of fluorescence, proportional to progressively increased Cd^2+^ concentrations, but also a perinuclear distribution (white arrow, 5 C), with some cup-shaped borders (yellow arrow, 5 C).

FC histograms show an increase in HNBO-DEN MFI in progressively Cd^2+^ polluted samples (Fig. 5D), for both hyalinocytes (red histograms) and granulocytes (green histograms).

The same investigations were performed after 1 h CdCl_2_ treatments: in hyalinocytes, high HNBO-DEN MFI values were collected in 1 mM CdCl_2_ treated cells (Fig. 5B). In granulocytes, after 1 h, the dye did not report the highest MFI values, apparently attesting to not reveal the highest Cd^2+^ content; however, this may reside in the strong increase of dead cells in 1 mM CdCl_2_ treated samples (Fig. 4E). CM analysis supported this interpretation, in particular for 1 mM concentration (Fig. 5B, C), in which the more fluorescent cells appeared spherical, without evidence of protrusions and with reduced dimensions, features typical of hyalinocytes. Such data requires further investigation (by deeply characterizing necrotic/apoptotic cells) and represent a problem to be addressed.

#### HNBO-DEN-NPs on mussel hemocytes: effect of cadmium exposure on hemocytes of *Mytilus galloprovincialis*

**Fig. 6:**
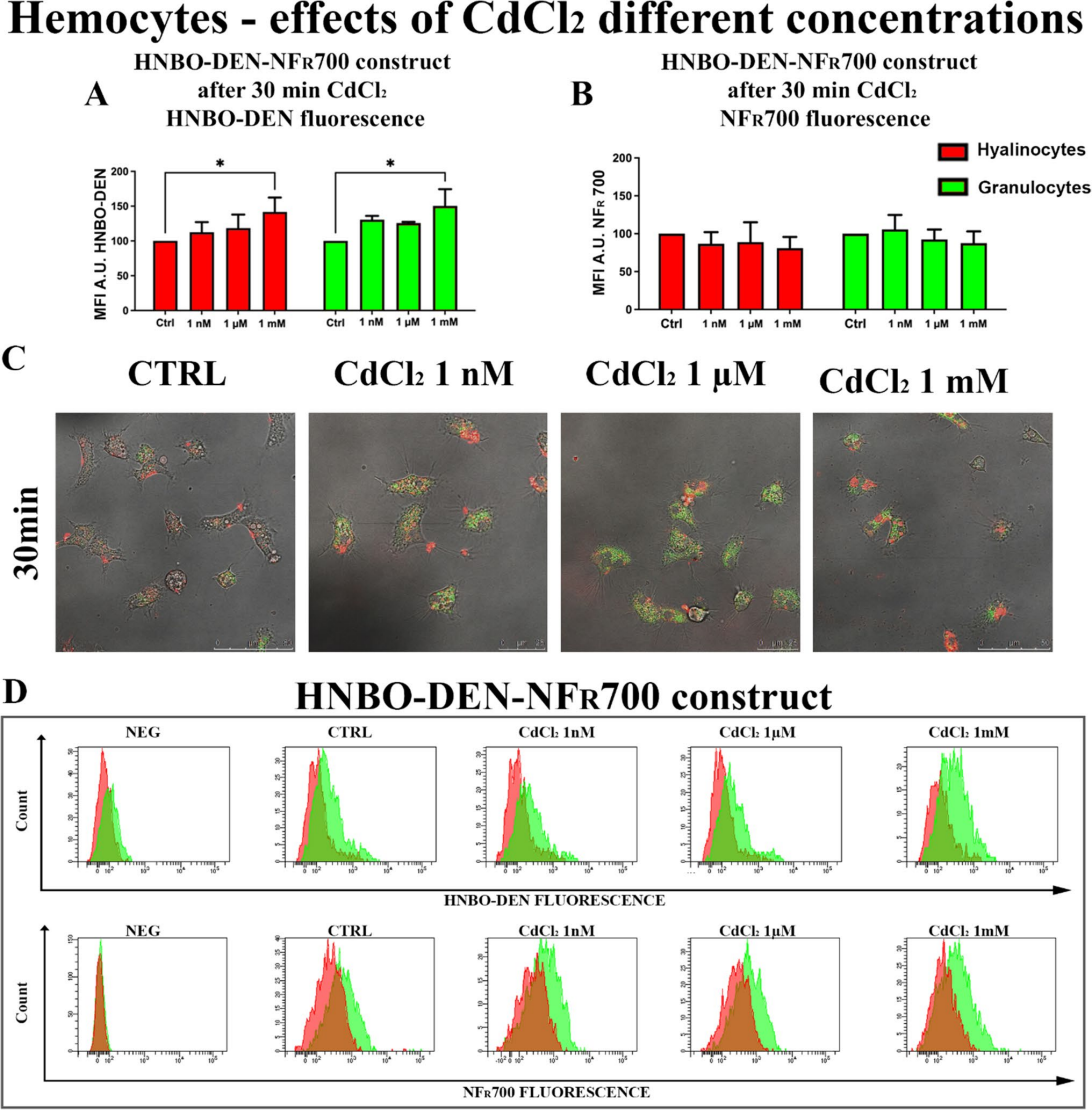
Hemocytes—CdCl_2_ increasing concentration: 30 min exposure.** A** Statistical histograms of HNBO-DEN MFI in hyalinocytes and granulocytes HNBO-DEN-NPs labelled at 30 min for control (CTRL) and treated cells with CdCl_2_ at different concentrations (1 nM, 1 µM, and 1 mM). **B** Statistical histograms of NF_R_700 MFI in hyalinocytes and granulocytes HNBO-DEN-NPs labelled at 30 min for control (CTRL) and treated cells with CdCl_2_ at different concentrations (1 nM, 1 µM, and 1 mM). **C** Single confocal optical sections of HNBO-DEN (green) and NPs (red) in *Mytilus galloprovincialis* hemocytes at 30 min for control (CTRL) and treated cells with CdCl_2_ at different concentrations (1 nM, 1 µM, and 1 mM). **D** Cytometric histogram overlay of HNBO-DEN fluorescence (above) and NF_R_700 fluorescence (below) in hyalinocytes (red) and granulocytes (green) for all conditions. Two-way ANOVA with Bonferroni’s multiple comparison revealed: *p < 0.05, **p < 0.01, ***p < 0.001

In the CdCl_2_ contamination hemolymph model, the HNBO-DEN-NPs construct was also tested. After 30 min, the dye-derived fluorescence occurred proportionally to the cadmium concentration in both hyalinocytes and granulocytes. This fluorescence increase was significant solely in cells treated with the 1 mM CdCl_2_ concentration (Fig. 6A).

A possible increase in HNBO-DEN fluorescence values due to NP “fluorescence contamination”, was also evaluated. For this reason, fluorescence emission at wavelength peaking around 519–525 nm was also calculated in cells stained with HNBO-DEN-unbound NPs (Figure S7A).

The results demonstrated that this emission by HNBO-DEN-unbound NPs (NPs alone) remained almost the same for all Cd^2+^ treatments; indeed, NF_R_700 specific emission (700 nm) did not appear to be proportional to the administered cadmium doses (Fig. 6B), supporting the idea that NP internalization process is not perturbed by the examined Cd^2+^ amounts.

In the CM images, a different localization of these two different fluorescence emissions (the green one-HNBO-DEN and the red one-NF_R_700) derived from the HNBO-DEN-NPs construct can be observed, particularly for hemocytes treated with the various Cd^2+^ concentrations after 30 min (Fig. 6C). The green fluorescence was weak and diffused in untreated hemocytes, whereas in treated hemocytes green fluorescence (revealing HNBO-DEN signal) became more intense and punctate. Red fluorescence (revealing NPs distribution) highlighted the same behavior. FC overlaid histograms (Fig. 6D) highlight the methodological approach in analyzing FC data files.

Since nanoparticles often converge in lysosomes because of the cellular endocytic pathway, through which they enter the cell, it is conceivable that the punctate distribution corresponds to lysosomes playing a crucial role in various functions, including the elimination of harmful substances, furthermore lysosomes can accumulate HMs, organic compounds and pharmaceuticals [56].

**Fig. 7:**
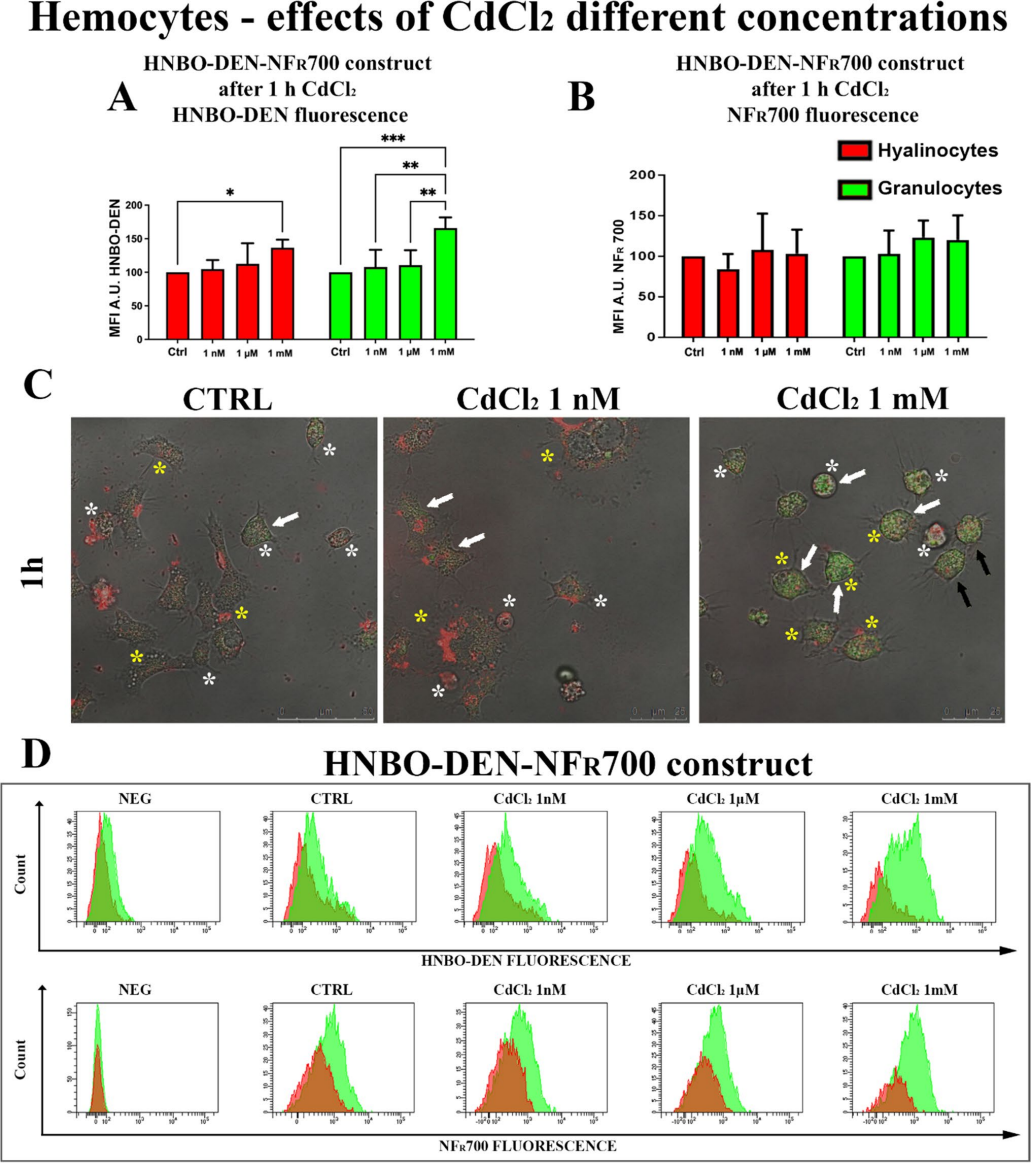
Hemocytes—CdCl_2_ increasing concentration: 1 h exposure.** A** Statistical histograms of HNBO-DEN MFI in hyalinocytes and granulocytes HNBO-DEN-NPs labelled after 1 h for control (CTRL) and treated cells with CdCl_2_ at different concentrations (1 nM, 1 µM, and 1 mM); **B** Statistical histograms of NF_R_700 MFI in hyalinocytes and granulocytes HNBO-DEN-NPs labelled after 1 h for control (CTRL) and treated cells with CdCl_2_ at different concentrations (1 nM, 1 µM, and 1 mM); **C** Single confocal optical sections of HNBO-DEN (green) and NPs (red) in *Mytilus galloprovincialis* hemocytes at 1 h for control (CTRL) and treated cells with CdCl_2_ at different concentrations (1 nM and 1 mM). White asterisks and yellow asterisks indicate hyalinocytes and granulocytes respectively; black arrows show cell morphologies changing and white arrows represent green cells fluorescence. **D** Cytometric histogram overlay of HNBO-DEN fluorescence (above) and NF_R_700 fluorescence (below) in hyalinocytes (red) and granulocytes (green) for all conditions. Two-way ANOVA with Bonferroni’s multiple comparison revealed: *p < 0.05, **p < 0.01, ***p < 0.001

After 1 h, the fluorescence derived from HNBO-DEN-NPs significantly increased in both hyalinocytes and granulocytes treated with 1 mM CdCl2 (Fig. 7A). As expected, after 1 h, the fluorescence emission at wavelength peaking around 519–525 nm was not perturbed by NPs (Fig. 7B), as previously reported in Figure S7B. Notably, in granulocytes after 1 mM Cd^2+^ treatment, it could be observed: (i) the rise in HNBO-DEN fluorescence, deriving from the net fluorescence of single cells (quantitated by FC in Fig. 7A) and (ii) the increase in the number of green-positive cells (white arrows in Fig. 7C). Indeed, microscopy highlights the morphological differences between hyalinocytes and granulocytes that, although still observable, become less evident due to a double, simultaneous process. This dual process is represented, on one hand, by a harmful effect of Cd^2+^ that inducing a progressive detachment and change in the shape and size of granulocytes (yellow asterisks), and on the other hand, a strong increase in hemocyte filopodial elongations, including hyalinocytes (black arrows), that has recently been observed [57] in mussels exposed to chemical pollution, indicating that stress can trigger this cellular response.

#### Combining data from both staining systems: can it be a useful approach?

1. The adopted systematic troubleshooting approach was the following: (1) Define the problem by clearly identifying it; (2) Analyze the problem to find the possible solution, step by step; (3) Find potential solutions and choose the most applicable/useful; (4) Adopt the solution to resolve the issue; and (5) Evaluate if the problem is fully resolved and detail the process. Translating this approach into the project, the first problem that needed to be solved was the cell compatibility of HNBO-DEN and the construct HNBO-DEN-NF_R_700, and their ability to detect intracellular cadmium levels: this issue started to be addressed for both staining systems, in a human cell line. Indeed, as a second step, we tested whether these systems could work on a classical in vitro model, mainly represented by HT-29 cells (with very low or virtually absent levels of Cd, under baseline control conditions) and on a more complex ex vivo model, represented by hemocytes from *Mytilus galloprovincialis* (in which some Cd amounts, absorbed from the mussel marine environment, may be physiologically present): this issue appears to have been addressed for both staining systems, although to different extents for the two cellular models.
2. To clarify some differences in the cellular response to cadmium emerged from the staining results of the mussel samples, confocal microscopy analyses were applied to confirm the quantitative data and render the application of these staining systems more informative; however, for HNBO-DEN intracellular distribution, further experiments are needed to deepen the organelle tropism of the dye.

**Fig. 8:**
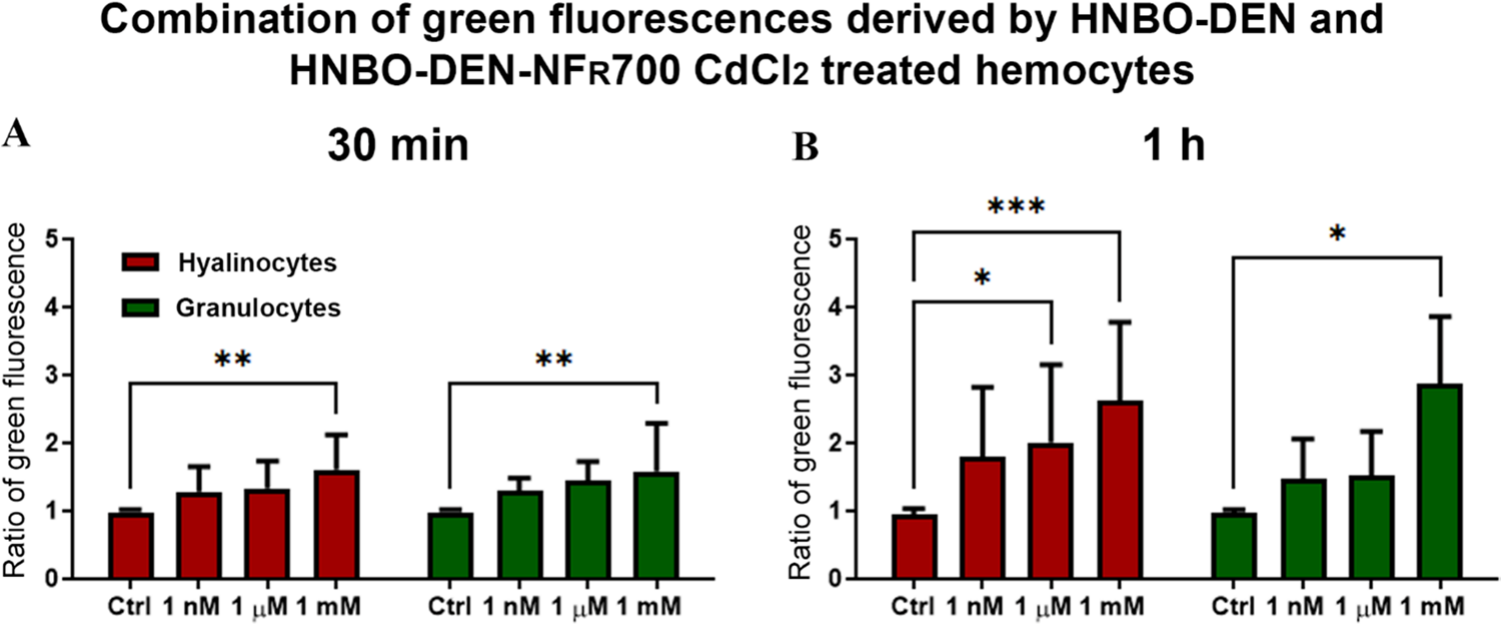
CdCl_2_ increasing concentration: Analyzing cadmium content through both staining systems**. A** Statistical histograms of ratio HNBO-DEN MFI derived by HNBO-DEN dye and HNBO-DEN-NPs construct in CdCl_2_ treated hemocytes after 30 min. **B** Statistical histograms of ratio HNBO-DEN MFI derived by HNBO-DEN dye and HNBO-DEN-NPs construct in CdCl_2_ treated hemocytes after 1 h. Two-way ANOVA with Bonferroni’s multiple comparison revealed: *p < 0.05, **p < 0.01, ***p < 0.001

Finally, a combination of Mean Fluorescence Intensity (MFI) data for dye fluorescence was established using the most complex model: ex vivo mussel hemocytes (Fig. 8). Graphs A and B highlight a visible proportionality between Cd concentrations and fluorescence intensity. While standard deviations increased—likely due to the integration of two detection systems within a complex biological matrix—a clear trend emerged across the different concentrations.

3. These combined data underscore the potential use of both HNBO-DEN and HNBO-DEN-NPs for cadmium detection, although the specific staining mechanisms remain only partially elucidated in the current study.

## Conclusion

We are normally exposed to low levels of Cd either at the workplace or by ingesting contaminated water and food [58]. In summary, Cd represents a persistent threat to human health due to its long biological half-life and primary entry through tobacco smoke and ingestion [58–60]. Furthermore, the use of marine mussels as bioindicators remains essential for monitoring the environmental accumulation of such anthropogenic trace metals in aquatic ecosystems [61–67]. In this framework, the newly developed ESIPT-based fluorescent chemosensor HNBO-DEN—consisting of a 2-(2-hydroxy-3-naphthyl)−4-methylbenzoxazole fluorophore linked to a N,N-bis(2-aminoethyl)amine receptor—proved highly effective for cadmium detection. Our findings demonstrate a robust, fluorescence increase (quite dose-dependent) in both HT-29 human intestinal cells and mussel hemocytes. Notably, the conjugation of the HNBO-DEN probe with NF_R_700 NPs preserved the dye sensing capability while providing critical diagnostic advantages: it enabled the precise tracking of nanoparticle internalization and allowed the discrimination of Cd accumulation between viable and damaged cells. In detail, with the employment of HNBO-DEN-NPs, it is important that viability will be finely checked and eventually integrated/combined with the ancillary infos descending by the application of the HNBO-DEN-NPs system. Both formulations emerge as powerful tools for Cd^2+^ and heavy metal monitoring, ensuring the maintenance of sample viability during analysis.

## Supplementary Information

Below is the link to the electronic supplementary material.

Supplementary file1 Document S1. Tables S1 and S2, Figures S1, S2, S3, S4, S5, S6, S7 and supplemental references. (PDF 2.94 MB)

## Data Availability

Requests for further information and resources should be directed to and will be fulfilled by the lead contacts, Mariele Montanari and Barbara Canonico (mariele.montanari@uniurb.it; barbara.canonico@uniurb.it).
