# Supplementary material for: Employment of the fluorescent probe hydroxy-naphthyl-methylbenzoxazole-based dye and its combination with fluorescent silica nanoparticles as potential heavy metal-targeting systems: results and troubleshooting in Cd-polluted environments
Source: Anal Bioanal Chem. 2026 Jun 8;418(15):4967–85. doi: 10.1007/s00216-026-06601-3 (PMC13388445; doi:10.1007/s00216-026-06601-3)
Supplement: Supplementary file 1 — Supplementary file1 Document S1. Tables S1 and S2, Figures S1, S2, S3, S4, S5, S6, S7 and supplemental references. (PDF 2.94 MB) [file 216_2026_6601_MOESM1_ESM.pdf]

SUPPLEMENTARY MATERIALS OF

Employment of the fluorescent probe hydroxy-naphthyl-methylbenzoxazole-based dye and its combination with fluorescent silica nanoparticles as potential heavy metal-targeting systems: results and troubleshooting in Cd-polluted environments

Daniele Lopez[1,2], Mariele Montanari[1*], Caterina Ciacci[1], Giovanna Panza[1], Eleonora Macedi[2], Daniele Paderni[2], Ludovica Di Fabrizio[1], Mattia Tiboni[1], Daniele Gori[3], Chiara Barattini[3], Angela Volpe[3], Alfredo Ventola[3], Stefano Papa[1], Vieri Fusi[2] and Barbara Canonico[1*]

[1]Department of Biomolecular Sciences (DISB), University of Urbino Carlo Bo, 61029 Urbino, Italy

[2]Department of Pure and Applied Sciences (DiSPeA), University of Urbino Carlo Bo, 61029 Urbino, Italy

[3]AcZon srl, 40050 Monte San Pietro, Italy

[*]Corresponding authors mariele.montanari@uniurb.it; barbara.canonico@uniurb.it

SUPPLEMENTARY MATERIALS AND METHODS

NP synthesis

Micelles play the role of small reactors where all reagents spontaneously arrange. Since the silane precursor (methyltrimethoxysilane or MTMS) is hydrophobic, after the addition of a basic catalyst (ammonia solution), the base-catalyzed hydrolysis and NP silica core condensation inside the micelle starts. PEG-Sil molecules arrange themselves with the hydrophilic chain towards the

aqueous environment and the hydrophobic silane head within the micelle, where it ends up being covalently bound to the silica matrix. This arrangement of the reagents leads to the formation of monodisperse core-shell NPs. The shell is composed of two different polyethylene glycols, H3CO-PEG-Sil and NH2-PEG-Sil. PEG provides NPs stability and solubility in water. The addition of an amino-terminal PEG makes it possible to conjugate the NPs after their synthesis. Amino functional groups could be directly conjugated to activated esters containing biomolecules or other organic compounds. They could also be easily transformed into carboxylic groups by reacting with a cyclic anhydride. The initial mixture may contain one or more hydrophobic dyes, in-house modified by adding a triethoxysilane group. Thanks to this feature, dyes enter the core of the micelles and covalently bind to the silica matrix. Once the reaction is over, several purification and clarification steps are applied (batchwise hydrophobic interaction chromatography, dialysis, high-speed centrifugation, serial filtrations) to remove the unreacted species. The resulting NPs are characterized by means of spectrophotometry, spectrofluorometry, Dynamic Light Scattering (DLS), fixed residue and zeta potential measurement. Since dyes form a covalent bond with the silica matrix, they are not released over time. NPs with two or more dyes (NanoTandem) emit light at wavelengths considerably longer than the absorbed one thanks to Förster Resonance Energy Transfer (FRET). To achieve high efficiency FRET, fluorophores are chosen such that the emission spectrum of the donor overlaps with the absorption spectrum of the acceptor. FRET efficiency strongly depends on the amount of each dye, donor/acceptor distance and donor/acceptor ratio. Controlling these three physicochemical parameters leads to FRET efficiency optimization. NPs with a single type of dye are called NanoChromes. In this case no FRET occurs, and the physicochemical features of the NPs are those of the included dye. In this case, controlling the dye concentration inside the particles is fundamental to prevent self-quenching phenomena, which occur at high dye concentrations. In both kinds of NPs, the silica matrix protects dyes from the external environment, thus increasing the stability of the dyes with time. The presence of PEG within the shell provides the functional groups that allow for the conjugation to other molecules.

HNBO-DEN and* NF_R700 *Silica Nanoparticle Conjugation

First 6 standards of known amine concentration are prepared by dissolving glycine in 100 mM Na2CO3/NaHCO3 pH 8.5 (reaction buffer or RB) (see Table S2). The NPs sample is prepared as follows: add 100 µL NPs + 400 µL RB + 500 µL TNBSA 1 mM in RB (10-fold sample dilution). The blank was prepared as follows: 500 µL of TNBSA and 500 µL of RB. The 6 standards, the sample and the blank were allowed to react for 2h at 40 °C, then 10 µL of HCl 37% w/w were

added and mixed well. 910 µL of the blank solution were mixed with 100 µL of fresh NFR700 and used to set the zero at 335 nm at the spectrophotometer. Then the absorbance of the sample and the standards was red. Standards absorbance and concentration were used to elaborate a calibration curve; sample data were interpolated to obtain amine concentration.

Intracellular Cadmium Detection: Comparative Analysis

HT-29 cells were treated with 500 µM $CdCl_2$ for 1 h. Following treatment, cells were detached using Trypsin-EDTA. To evaluate and compare their efficiency in detecting intracellular cadmium, cells were separately labeled with HNBO-DEN (500 nM) or Leadmium™ Green AM (2 µM). Staining was performed for 20 min at room temperature (RT) in the dark, and cells were immediately acquired via flow cytometry.

Table S1: Applications of fluorescent probes for HMs detection in the environmental field.

PROBE	***ANALYTES***	***LOD***	***WORKING SOLUTION***	***APPLICATION***	***REF.***
**1 O**	**CD^{2+}**	**5.74×10^{-7} M**	**THF**	**WATER**	[1]
**PIS**	**CD^{2+}**	**2.10×10^{-8} M**	**ACN/HEPES (10 MM, PH = 7.4) (1:4, *V*/*V*)**	**ZEBRAFISH**	[2]
**PMPA**	**CD^{2+}**	**0.12 MM**	**ACN**	**WATER**	[3]
**PROBE 1**	**CD^{2+}**	**0.055 MM**	**ACN**	**WATER, BEAN SPROUTS**	[4]
**QTPY**	**CD^{2+}**	**3 X 10^{-8}M**	**DMF/H2O (4:6, *V*/*V*)**		[5]
**L**	**CD^{2+}**	**14.8 NM**	**ACN/H2O (8:2, *V*/*V*)**	**WATER**	[6]
**DDTQ**	**CD^{2+}**	**126 NM**	**H2O**	**CELL IMAGING**	[7]
**1A**	**CD^{2+}**	**5.84×10^{-8} M**	**HEPES (20 MM, ACN/H2O, 3:7, *V*/*V*, PH = 7.0)**		[8]
**1**	**CD^{2+}**	**0.114 MM**	**THF/H2O (1:1, *V*/*V*)**		[9]
**NIS**	**CD^{2+}**	**3.87×10^{-7} M**	**HEPES (ETOH/H2O = 9:1, *V*/*V*, PH = 7.4)**	**ZEBRAFISH**	[10]

BPC	CD^{2+}	1.05×10^{-8} M	ACN/TRIS-HCL (3:2, *V*/*V*, PH = 7.4)	WATER	10
[PTZ-SB][BR]	CD^{2+}	3.8×10^{-7} M	THF/H2O (1:9, *V*/*V*)	WATER	11
IHL	CD^{2+}	0.4×10^{-10} M	DMSO/H2O (9:1, *V*/*V*)	ZEBRAFISH	12
L	CD^{2+}	0.03 PPM	ACN/H2O (4:1, *V*/*V*) (1:9, *V*/*V*)		13
PY	CD^{2+}	0.09 MM	MEOH (PH = 7, PBS)	WATER	14
CM 1	CD^{2+}	19.25 NM	H2O	WATER	15
PM	CD^{2+}	53 NM	H2O/DIOXANE (1/19, *V*/*V*)	WATER, RICE	16
L1	CD^{2+}		MECN/H_2O 4:1 V/V (MOPS $1.0X10^{-2}$ M, PH 7.4)	SOILS	17
HNBO (IN ARRAY)	CD^{2+}	0.0013 MG/L	HEPES PH 7.5	NATURAL WATER SAMPLES	18
L3	MG^{2+}	0.6 NM	DMSO + 1.5% WATER	DRINKING WATER	19
L3	MG^{2+}, CD^{2+}		DMSO OR ACN + 1.5% WATER; HEPES PH 7.4		20

Table S2: Standards composition for the calibration curve.

STANDARD	VOLUME OF GLY SOLUTION 100 µM (µL)	VOLUME OF RB (µL)	VOLUME OF TNBS (µL)	[GLY] µM
6	400	100	500	100
5	250	250	500	62,5
4	200	300	500	20
3	100	400	500	10
2	50	450	500	5

**1**	**20**	**480**	**500**	**2**
**BLANK**	**0**	**500**	**500**	**0**

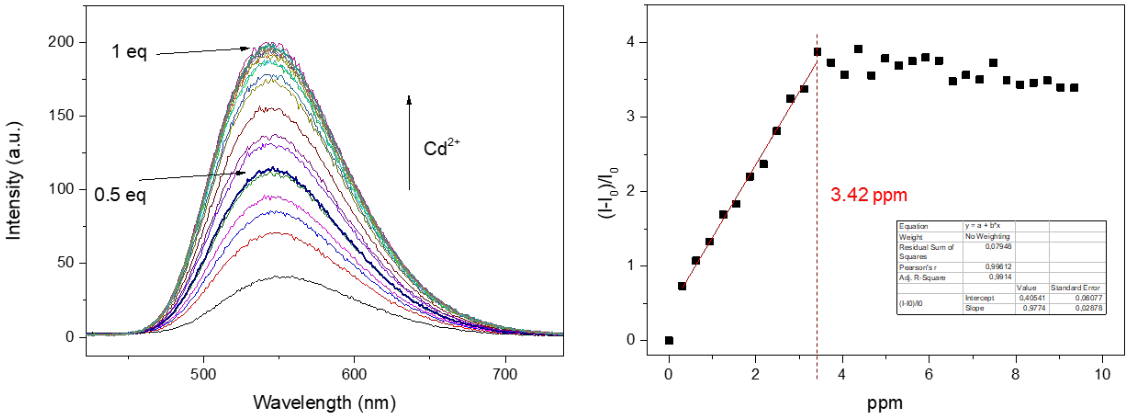

Figure S1 Left: Emission spectra of HNBO-DEN upon addition of Cd^{2+} up to 3 equiv. Right: Normalized intensity as a function of ppm of Cd^{2+} along with linear fit. [HNBO-DEN] = $1.0 \cdot 10^{-5}$ M.

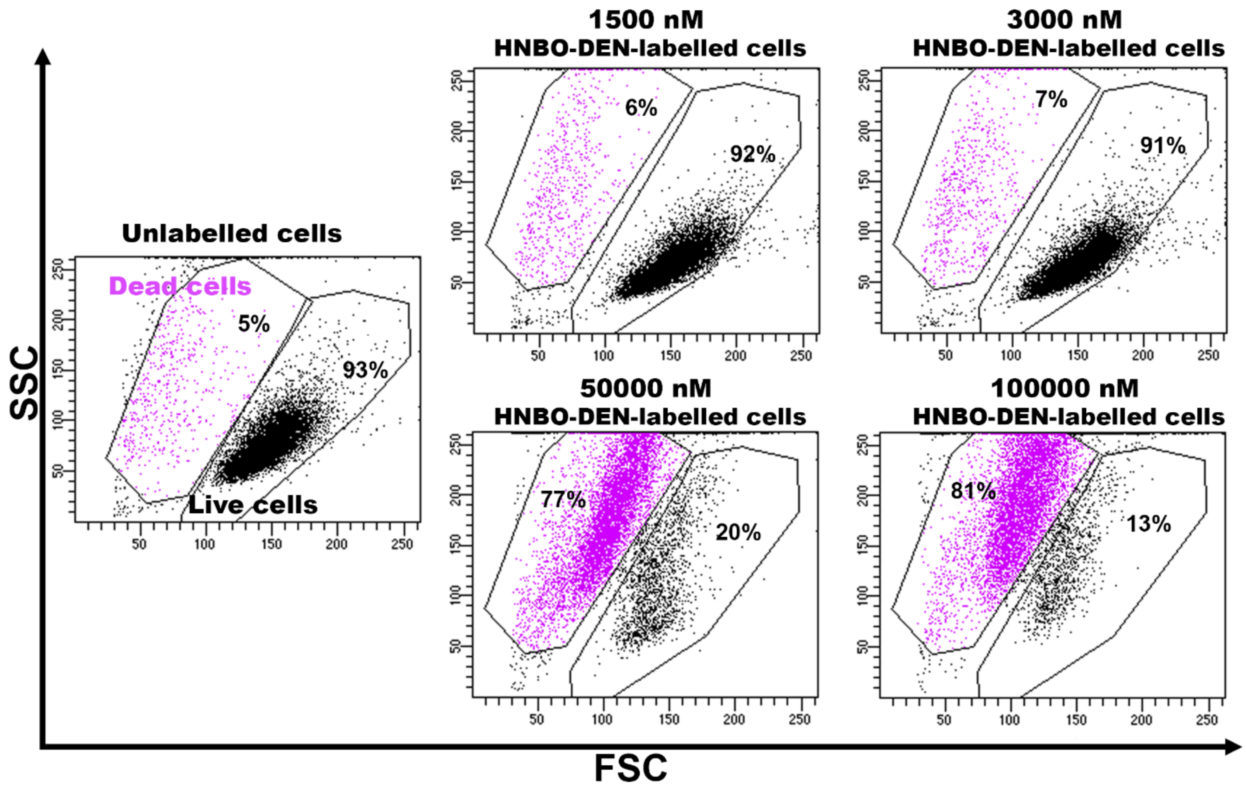

Figure S2: Evaluation of HNBO-DEN dye cytotoxicity via flow cytometry. Representative flow cytometry forward scatter (FSC) versus side scatter (SSC) plots are shown for cells labeled with varying concentrations of the HNBO-DEN dye (1500 nM to 100000 nM). The gating strategy used to differentiate the primary populations in live cells (black population) and the dead cells (magenta population. The subsequent panels display how increasing concentrations of HNBO-DEN affect increase in the percentage of dead cells.

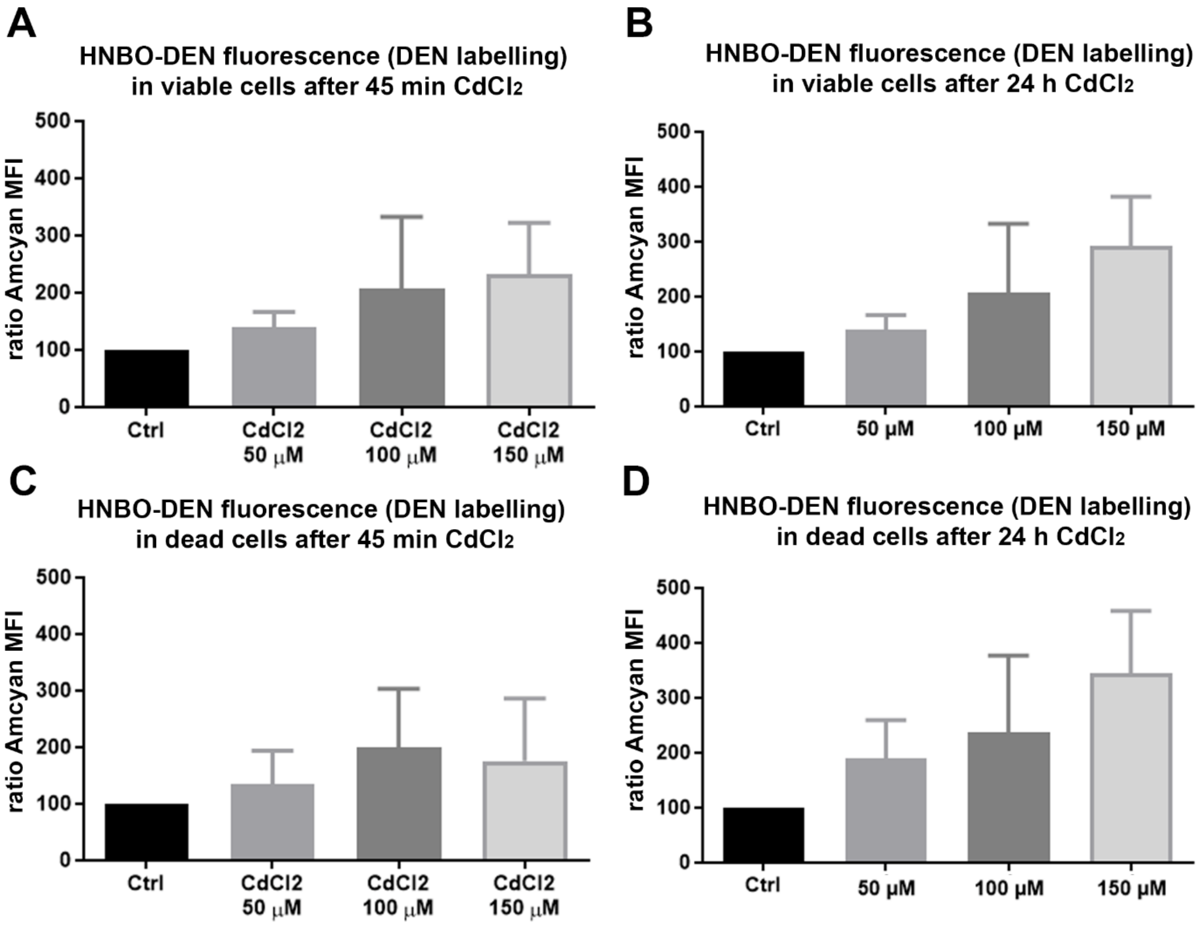

Figure S3. Evaluation of HNBO-DEN dye Amcyan fluorescence in viable and dead cells after CdCl2 treatment. (A, B) Statistical histograms show the ratio of amcyan MFI in viable cells treated with different concentrations of $CdCl_2$ (50 μM, 100 μM, and 150 μM) for 45 minutes (A) and 24 hours (B). (C, D) Statistical histograms show the ratio of amcyan MFI in dead cells treated with different concentrations of $CdCl_2$ (50 μM, 100 μM, and 150 μM) for 45 minutes (C) and 24 hours (D).

A Comparison of Methods for Metal Detection in Biological Samples

Method	Type	Advantages	Disadvantages	Live Cell Imaging?	Common Dyes / Probes
Fluorescent Probes (Ajavakom et al. 2026, Panza et al. 2025)	Chemical	High sensitivity; Real-time imaging; High spatial resolution	Potential toxicity; Interference from other metals (e.g., Zn^{2+})	Yes	HNBO-den, Leadmium Green, FluoZin-3, Rhodamine
Protein Biosensor (He et al. 2023)	Bio/Chemical	Superior specificity; Targetable to organelles (e.g., mitochondria)	Requires transfection; Weaker signal than small molecules	Yes	FRET-based sensors, GFP variants
ICP-MS (Zai et al. 2023)	Physical	Extreme quantitative accuracy; Ultra-trace detection	Destructive (cell lysis); High cost; No spatial info	No	N/A (elemental analysis of extracts)
XRF/ Nano-XRF (Tian et al. 2022)	Physical	Metal mapping without stains	Requires Synchrotron; Complex prep; Rare equipment	Limited	N/A (X-Ray fluorescence)
EM/EDX (Mutabaruka and Rana 2025)	Physical	Highest morphological resolution; Subcellular visualization	Low trace sensitivity; Invasive prep	No	N/A

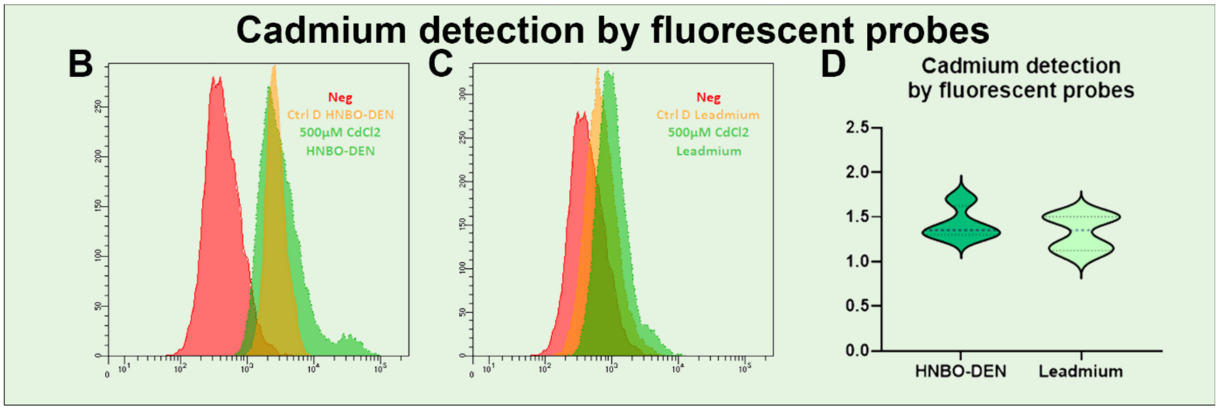

Figure S4: Methodological overview of metal detection techniques and experimental validation of cadmium-selective fluorescent probes A): Table highlighting the different methods for metal detection in biological samples. Image generated using FigureLabs and subsequently modified by the author (figurelabs.ai) **B):** Histogram overlays related to unlabelled HT-29 cells (Red neg), labelled unpolluted HT-29 cells (Orange Ctrl D HNBO-Den), labelled polluted HT-29 cells (Green 500 □M $CdCl_2$ HNBO-DEN); **C):** Histogram overlays related to unlabelled HT-29 cells (Red neg), labelled unpolluted HT-29 cells (Orange Ctrl Leadmium), labelled polluted HT-29 cells (Green 500 □M $CdCl_2$ Leadmium); **D):** Comparison between ratio MFI treated/MFI untreated for HNBO-DEN and Leadmium, it measures assay sensitivity (how many times the signal increases in response to cadmium).

Figure 4 References:

Ajavakom, Anawat, et al. 2026 "Fluorescent Probes for Heavy Metal Detection: A Review of Emerging Trends and Advances in Sensing Zinc, Cadmium, and Mercury Ions." Sensors International: 100374. https://doi.org/10.1016/j.sintl.2026.100374

Panza, Giovanna, et al. 2025 "Hepatopancreatic cells of Armadillidium vulgare: an integrated, flow cytometric-based biomarker approach to unravel the soil ecological disturbance." Science of the Total Environment 996: 180135. https://doi.org/10.1016/j.scitotenv.2025.180135P

He, Mei-Ying, et al. 2021 "Sensitive and specific cadmium biosensor developed by reconfiguring metal transport and leveraging natural gene repositories." ACS sensors 6.3: 995-1002. https://doi.org/10.1021/acssensors.0c02204

Zai, Yinghan, et al. 2023 "Development of a new detection method for cadmium in marine phytoplankton based on single cell inductively coupled plasma mass spectrometry." Spectrochimica Acta Part B: Atomic Spectroscopy 209: 106801. https://doi.org/10.1016/j.sab.2023.106801

Tian, Xiaosong, et al. 2022 "Identification of heavy metal pollutants and their sources in farmland: an integrated approach of risk assessment and X-ray fluorescence spectrometry." Scientific Reports 12.1: 12196. https://doi.org/10.1038/s41598-022-16177-4

Mutabaruka, Michel, and Aditya Rana 2025 "Use of EDS/EDX to evaluate heavy metals pollution in water sources." Next Sustainability 5: 100082. https://doi.org/10.1016/j.nxsust.2024.100082

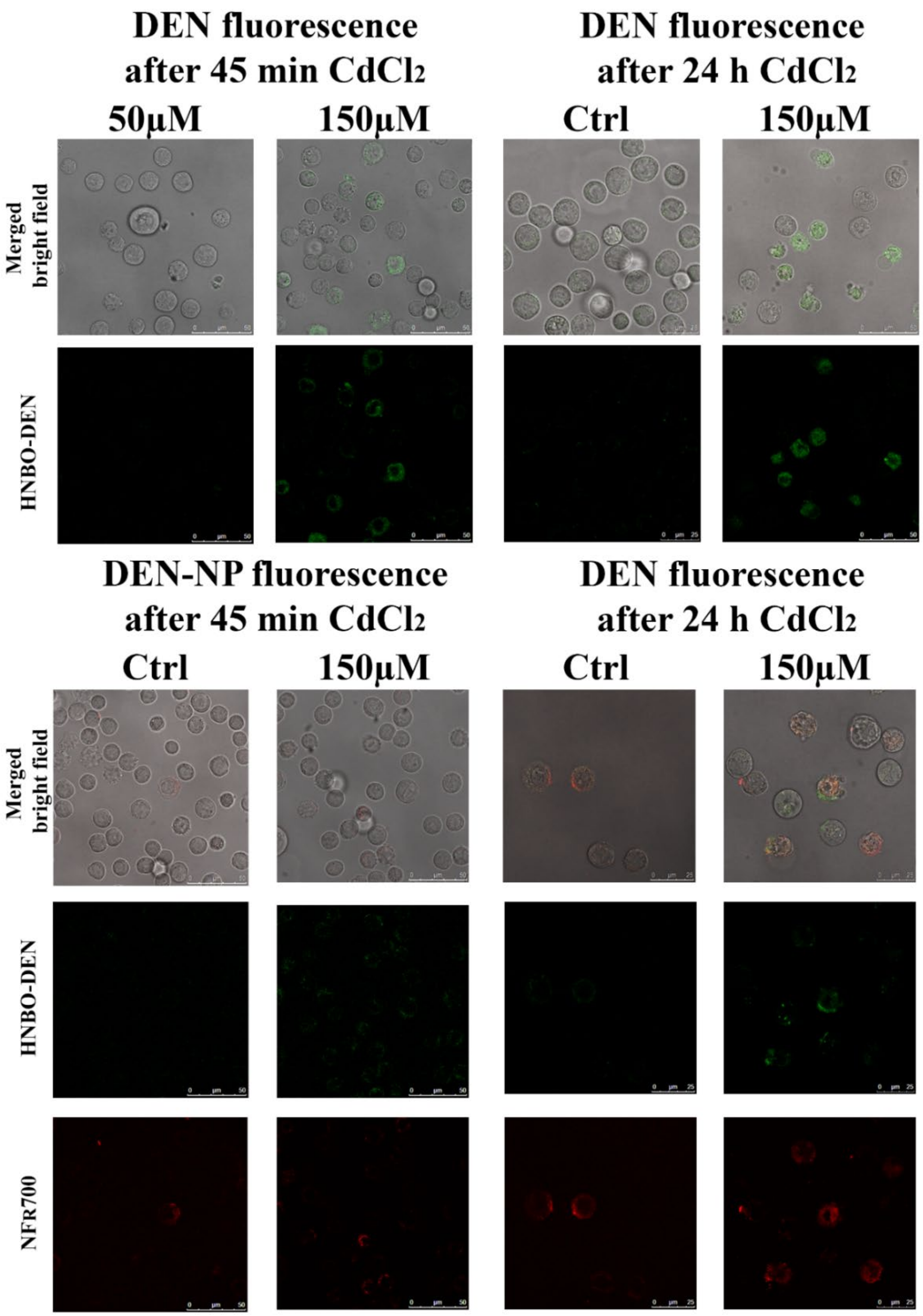

Figure S5: Confocal microscopy images of cells after exposure to $CdCl_2$. The top rows show HNBO-DEN fluorescence (green channel) and merged bright-field images, indicating the intracellular localization of the probe. The bottom rows display the fluorescence of HNBO-DEN-NP construct HNBO-DEN (green channel) and NP (NFR700, red channel), illustrating the different uptake and/or processing mechanisms of the free dye versus the nanoparticle formulation under varying conditions of $CdCl_2$ exposure.

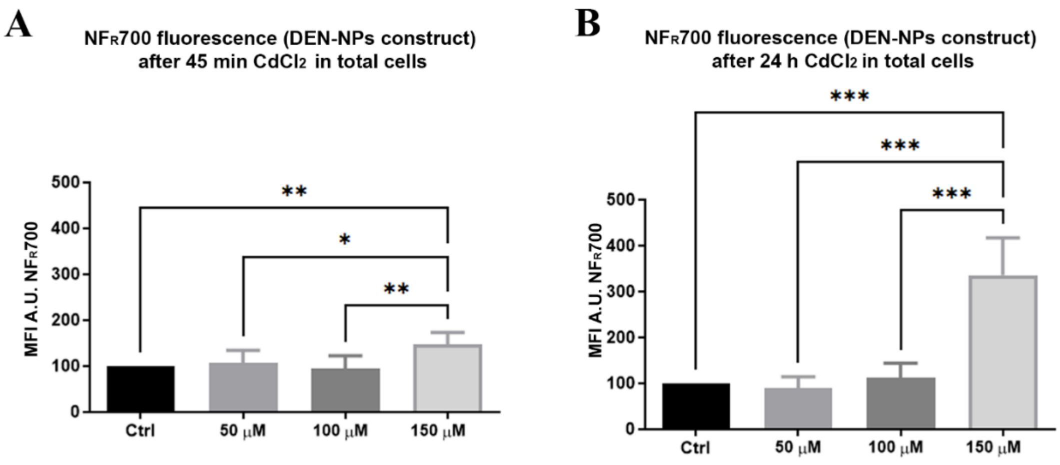

Figure S6: A) Statistical histograms of NFR700 MFI in total HT-29 cells at 45 min for Control (CTRL) and treated cells with CdCl2 at different concentrations (50μM, 100μM and 150μM); B) Statistical histograms of NFR700 MFI in total HT-29 cells at 24h for Control (CTRL) and treated cells with CdCl2 at different concentrations (50μM, 100μM and 150μM). One-way ANOVA with Bonferroni's multiple comparison revealed: * = $p < 0.05$, ** = $p < 0.01$, *** = $p < 0.001$.

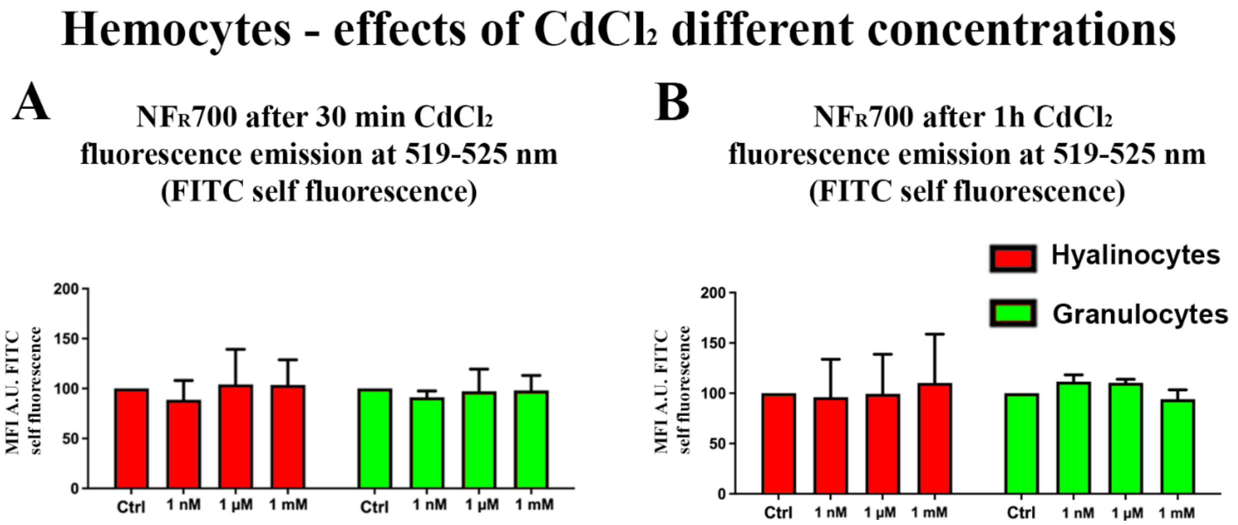

Figure S7: A) Statistical histograms of FITC self-fluorescence in Hyalinocytes and Granulocytes NFR700 labelled after 30 min treatment with CdCl2 at different concentrations (1nM, 1μM and 1mM); B) Statistical histograms of FITC self-fluorescence in Hyalinocytes and Granulocytes NFR700 labelled after 1 h treatment with CdCl2 at different concentration (1nM, 1μM and 1mM).

SUPPLEMENTARY REFERENCES

1. Zhang SL, Xu JJ, Shen Y, Wu T, Wang Y, Zhao X, Chen HY. Nucleolin-targeted ratiometric fluorescent carbon dots with a remarkably large emission wavelength shift for precise imaging of cathepsin B in living cancer cells. Anal Chem. 2021; 93:4042–4050. https://doi.org/10.1021/acs.analchem.0c05046.

2. Radiul SM, Hazarika S. Variation of Stokes Shift and Peak Wavelength Shift as a Sensing Probe for Detection of Lead in Water Using Laser Induced Fluorescence Resonance Energy Transfer J Fluoresc. 2021; 31: 889–896. https://doi.org/10.1007/S10895-021-02689-1.

3. Zhang Y, Chen L, Yang J, Zhang Y, Yuan MS. An “OR-AND” logic gate based multifunctional colorimetric sensor for the discrimination of Pb2+ and Cd2+. Spectrochim. Acta Part A Mol. Biomol. Spectrosc. 2020; 232:118163. https://doi.org/10.1016/j.saa.2020.118163.

4. Punithakumari G, Wu SP, Velmathi S. Highly Selective Detection of Cr3 + Ion with Colorimetric & Fluorescent Response Via Chemodosimetric Approach in Aqueous Medium. J Fluoresc. 2018; 28:663–670. https://doi.org/10.1007/S10895-018-2228-1.

5. Minhaz A, Anwar A, Ahmad I, Khattak R, Yaseen M, Ahmed F, Shah MR, Khan NA, Ishaq M. A flexible Schiff base probe for spectrophotometric detection of chromium (III). Int. J. Environ Sci Technol. 2019; 16:5577–5584. https://doi.org/10.1007/S13762-018-2103-2.

6. Liu Y, Li L., Zhang Y, Sheng M, Wang Y, Xing Z, Yang L, Yue M, Fu Y, Ye F. A novel functional fluorescent probe based on a pyrene derivative for the detection of multiple pollutants. J Mol Liq. 2023; 382:121888. https://doi.org/10.1016/j.molliq.2023.121888.

7. Liu Y,Yang F, Wei K, Kang M, Liu P, Yang X, Pei M, Zhang G. 5-(Thiophene-2-yl)oxazole derived “off-on-off” fluorescence chemosensor for sequential recognition of In3+ and Cr3+ ions. J Photochem Photobiol A Chem. 2023; 437:114464. https://doi.org/10.1016/j.jphotochem.2022.114464.

8. Wang Z, Zheng C, Xu D, Liao G, Pu S. A fluorescent sensor for Zn2+ and Cd2+ based on a diarylethene derivative with an indole-2-methylhydrazone moiety. J Photochem Photobiol A Chem. 2022; 424:113634. https://doi.org/10.1016/J.JPHOTOCHEM.2021.113634.

9. Ma J, Dong Y, Yu Z, Wu Y, Zhao Z. A pyridine based Schiff base as a selective and sensitive fluorescent probe for cadmium ions with “turn-on” fluorescence responses. New J Chem. 2022; 46:3348–3357. https://doi.org/10.1039/d1nj05919j.

10. Rout K, Manna AK; Sahu M, Mondal J, Singh SK, Patra GK. Triazole-based novel bis Schiff base colorimetric and fluorescent turn-on dual chemosensor for Cu2+ and Pb2+: application to living cell imaging and molecular logic gates. RSC Adv. 2019; 9:25919–25931. https://doi.org/10.1039/C9RA03341F.

11. SongH, and Zhang Z. A quinoline-based ratiometric fluorescent probe for discriminative detection of Zn2+ and Cd2+ with different binding modes, and its Zn2+ complex for relay sensing of pyrophosphate and adenosine triphosphate. Dye Pigment. 2019; 165:172–181. https://doi.org/10.1016/J.dyepig.2019.02.011.

12. Xiao Y, Ma L, Li D, Liu L, Wang H. Preparation 4’-Quinolin-2-yl-[2, 2’; 6’, 2”] terpyridine as a ratiometric fluorescent probe for cadmium ions and zinc ions in aqueous. J Photochem Photobiol A Chem. 2020; 399:112613. https://doi.org/10.1016/J.jphotochem.2020.112613.

13. René W, Lenoble V, Chioukh M, Branger C. A turn-on fluorescent ion-imprinted polymer for selective and reliable optosensing of lead in real water samples. Sensors Actuators B Chem. 2020; 319:128252. https://doi.org/10.1016/j.snb.2020.128252.

14. Isaad J, Malek F, Achari AE. Colorimetric and fluorescent probe based on coumarin/ thiophene derivative for sequential detection of mercury(II) and cyanide ions in an aqueous medium. J Mol Struct. 2022; 1270:133838. https://doi.org/10.1016/j.molstruc.2022.133838.

15. Huang MX,Lv CH, Huang QD, Lai JP, Sun H. A novel and fast responsive turn-on fluorescent probe for the highly selective detection of Cd2+ based on photo-induced electron transfer. RSC Adv. 2019; 9:36011–36019. https://doi.org/10.1039/c9ra06356k.

16. Zhang Z, Yuan S, Wang E.A Dual-Target Fluorescent Probe with Response-Time Dependent Selectivity for Cd2+ and Cu2+. J Fluoresc. 2018; 28: 1115–1119. https://doi.org/10.1007/S10895-018-2274-8.

17. Garau A, Lvova L, Macedi E, Ambrosi G, Aragoni MC, Arca M, Caltagirone C, Coles SJ, Formica M, Fusi V.N2S2 pyridinophane-based fluorescent chemosensors for selective optical detection of Cd2+ in soils. New J Chem. 2020; 44:20834–20852. https://doi.org/10.1039/d0nj03858j.

18. Lvova L, Caroleo F, Garau A, Lippolis V, Giorgi L, Fusi V, Zaccheroni N, Lombardo M, Prodi L, Natale CD et al. A fluorescent sensor array based on heteroatomic macrocyclic fluorophores for the detection of polluting species in natural water samples. Front Chem. 2018; 6:375396. https://doi.org/10.3389/FCHEM.2018.00258.

19. Paderni D, Macedi E, Lvova L,Ambrosi G, Formica M, Giorgi L, Paolesse R, Fusi V. Selective Detection of Mg2+ for Sensing Applications in Drinking Water. Chem - A Eur J. 2022; 28. https://doi.org/10.1002/chem.202201062.

20. Paderni D, Lopez D, Macedi E, Ambrosi G, Ricci A, Palazzetti E, Giorgi L, Formica M, Fusi V. (Solvent induced selective response to metal ions of three HNBO-based chemosensors. Inorganica Chim Acta. 2023; 549: 121400. https://doi.org/10.1016/j.ica.2023.121400.
